# Effect of dexamethasone on newborn survival at different administration-to-birth intervals: A secondary analysis of the WHO ACTION (Antenatal CorticosTeroids for Improving Outcomes in Preterm Newborn)-I trial

**DOI:** 10.1016/j.eclinm.2022.101744

**Published:** 2022-11-14

**Authors:** WHO ACTION Trials Collaborators, Olufemi T. Oladapo, Joshua P. Vogel, Gilda Piaggio, My Huong Nguyen, Fernando Althabe, Rajiv Bahl, Suman P.N. Rao, Ayesha De Costa, Shuchita Gupta, Abdullah H. Baqui, Mohammod Shahidullah, Saleha Begum Chowdhury, Shivaprasad S. Goudar, Sangappa M. Dhaded, Zahida P. Qureshi, Fredrick Were, John Kinuthia, Adejumoke I. Ayede, Olubukola A. Adesina, Oluwafemi Kuti, Ebunoluwa A. Adejuyigbe, Shabina Ariff, Sajid B. Soofi, Lumaan Sheikh, Jose Carvalho, Vania A. Nilsson, Luciana Abreu

## Abstract

**Background:**

The WHO ACTION-I trial demonstrated that dexamethasone significantly reduced neonatal mortality when administered to women at risk of early preterm birth in low-resource countries. We conducted a secondary analysis to determine how these benefits can be optimised, by evaluating the effect of dexamethasone compared to placebo on newborn mortality and severe respiratory distress outcomes at different administration-to-birth intervals, and identifying the interval with the greatest benefits.

**Methods:**

The WHO ACTION-I trial was a multi-country, individually-randomised, parallel-group, double-blind, placebo-controlled trial. It was conducted in 29 hospitals across Bangladesh, India, Kenya, Nigeria, and Pakistan. Women with a viable singleton or multiple pregnancy who presented to participating hospitals at a gestational age of 26 weeks 0 days–33 weeks 6 days and who were at risk of imminent preterm birth were eligible. In this secondary analysis, 2638 women and their newborns treated with single course of dexamethasone or placebo were analysed. Multivariate logistic regression was used to assess the effect of dexamethasone versus placebo on neonatal death, stillbirth or neonatal death, and severe respiratory distress at 24 h and at 168 h, by administration-to-birth interval (from 0 through 28 days), adjusting for gestational age at first dose. We used relative risks to identify the administration-to-birth interval with the greatest benefits of dexamethasone compared to placebo on the newborn outcomes.

**Findings:**

Between 24 December 2017 and 21 November 2019, 2852 women and their 3070 babies were enrolled in the WHO ACTION-I trial; 1332 women (1464 babies) in the dexamethasone group and 1306 women (1440 babies) in the placebo group were included in this secondary analysis. Neonatal mortality risk was lower with increasing time between initiating dexamethasone and birth, achieving peak mortality reduction by days 13 and 14 and then diminishing as the interval approached 28 days, regardless of gestational age at administration. For other outcomes, the overall pattern of risk reduction extending into the second week was consistent with that of neonatal death.

**Interpretation:**

In women at risk of preterm birth prior to 34 weeks’ gestation, the neonatal benefits of antenatal dexamethasone appear to increase with longer administration-to-birth intervals than previously thought. This knowledge can support clinical assessment and estimation of the risks of adverse preterm newborn outcomes at the time of birth, and the potential benefits of antenatal dexamethasone treatment for a known administration-to-birth interval.

**Funding:**

Bill and Melinda Gates Foundation; World Health Organization.

**Research in context**

**Evidence before this study**

We conducted a systematic search of PubMed, Embase, CINAHL, Cochrane Library and Global Index Medicus (using search terms and synonyms for “pregnancy” and “corticosteroid” and “duration”), with no date or language restrictions to identify randomised and non-randomised studies where newborn outcomes were reported for different antenatal corticosteroid administration-to-birth intervals. The review identified 59 studies (11 trials and 48 observational studies), largely from high-income country settings, and there was considerable heterogeneity in study populations, administration-to-birth intervals used, and conclusions. It was not possible to identify an optimal interval from existing evidence.

**Added value of this study**

To our knowledge, this is the first analysis of an antenatal corticosteroid efficacy trial that has identified further reduction in the relative risk of neonatal mortality with longer dexamethasone administration-to-birth intervals. The relative risk of severe neonatal respiratory distress at 24 h and at 168 h showed similar patterns although not as remarkable as that of neonatal death. This analysis goes beyond previous studies by using administration-to-birth interval as a continuous (rather than categorical) variable, and using multivariate modelling to account for the confounding effects of gestational age and other important maternal and newborn factors. We developed a practical tool for potential clinical application of these findings.

**Implications of all the available evidence**

The neonatal mortality reduction benefits from antenatal dexamethasone for women at risk of early preterm birth might be substantially higher with longer administration-to-birth intervals than generally thought, regardless of gestational age at the time of initiating treatment. Our findings question the scientific basis of clinical recommendations that are based upon the premise that ACS are not effective and even harmful after 7 days following initiation of treatment. The new knowledge generated from this study can be used to support clinical assessment and estimation of the risks of adverse preterm newborn outcomes at the time of birth, as well as the potential benefits of ACS treatment for a known administration-to-birth interval (such as in the context of provider-initiated preterm birth).

## Introduction

Antenatal administration of corticosteroids to women at risk of preterm birth has been shown to improve outcomes for preterm newborns. The 2020 update of the Cochrane review on efficacy of antenatal corticosteroids (ACS) demonstrated that a single course of dexamethasone or betamethasone prior to 34 weeks’ gestation confers significant reductions in neonatal mortality, and severe morbidities, without causing maternal or newborn harms.^1^ While previous trials on the efficacy of ACS were largely conducted in high-income countries,^2^ the recently published WHO ACTION (**A**ntenatal **C**orticos**T**eroids for **I**mproving **O**utcomes in preterm **N**ewborn)-I trial, which was conducted in hospitals across five low-resource countries, provide reassurance on the clinical benefits of ACS when used for women at risk of early preterm birth. The trial reported a substantial reduction in the risks of neonatal mortality alone and stillbirth or neonatal mortality, without increasing the risk of maternal or neonatal infection,^3^ and thus laid to rest the controversies surrounding the safety and efficacy of ACS in low-resource countries.

Now that the benefits of ACS administration in low-resource countries have been established, there is a need to determine how these benefits can be optimised in clinical practice. One important consideration is the potential relationship between the time interval from administration of ACS to birth and how the interval might affect the neonatal benefits of ACS. In contemporary obstetric practice, it is generally accepted that there is a minimum and a maximum time of in-utero “fetal exposure” to ACS to achieve clinical benefits and avoid harms. While this notion is reasonable from a scientific standpoint (particularly based on findings from animal studies), what constitutes the best (or optimal) time interval to maximise clinical benefits of ACS remains unclear. Several organisations, including WHO, recommend that administration of ACS should target eligible women with a high likelihood of preterm birth within 7 days of starting treatment, but the quality of the underlying evidence is low.^4^, ^5^, ^6^ Although the timing of provider-initiated preterm birth via labour induction or caesarean section can be reasonably anticipated, accurate prediction of the timing of spontaneously-initiated preterm birth even among those at highest risk is clinically difficult. Hence, adherence to current ACS recommendations regarding administration-to-birth interval remains practically challenging.

Previous studies exploring the association between time from ACS administration-to-birth and newborn health outcomes have generally been inconsistent in their findings. For example, a study in Canada by Melamed et al.^7^ reported that maximum benefits were observed when ACS was administered 1–7 days prior to birth. In contrast, an observational study by Norman et al.^8^ across several European countries reported that ACS were associated with a rapid decline in infant mortality even over a short period (less than 12 h) between administration and birth. Though the greatest reductions were observed at an interval between 18 and 36 h, the mortality benefits appeared to persist beyond 3 weeks. Other studies from high-income countries have also reported that benefits of ACS are time-related, though the intervals associated with improved outcomes are not consistent across studies.^7^^,^^9^, ^10^, ^11^ It is unclear whether these variations were the results of heterogeneity in clinical practice protocols, characteristics of the study populations, outcome measures, or inherent biases in the study designs. Many studies had pre-defined an interval between 24 h and 7 days as the “optimal interval” without analysing and reporting the time points associated with greatest risk reductions for specified adverse outcomes, or accounting for important confounders such as gestational age at the time of ACS administration. To address these shortcomings and provide data from low-resource country contexts, we conducted a secondary analysis of the WHO ACTION-I trial to evaluate the effect of dexamethasone compared to placebo on neonatal mortality and severe respiratory morbidity outcomes, by the administration-to-birth interval, in order to identify the interval with the greatest clinical benefits (i.e., an optimal interval).

## Methods

### Study design and participants

The design of the WHO ACTION-I trial has been described previously in the study protocol and primary findings.^3^^,^^12^ In brief, WHO ACTION-I trial was a multi-country, multi-centre, individually-randomised, parallel-group, double-blind, placebo-controlled trial. The trial was conducted in 29 secondary and tertiary level hospitals across Bangladesh, India, Kenya, Nigeria, and Pakistan. The trial protocol was approved by ethics committees and regulatory agencies for each country and participating hospital and the WHO Ethics Review Committee and registered prior to commencement (Australia and New Zealand Clinical Trials Registry number ACTRN12617000476336; Clinical Trials Registry-India number, CTRI/2017/04/008326). The trial was conducted and reported in accordance with Consolidated Standards of Reporting Trials (CONSORT) guidelines.^13^ All participants were randomized between December 2017 and November 2019, and the trial was stopped early due to evidence of benefit.^3^^,^^12^

Women with a viable singleton or multiple pregnancy who presented to participating hospitals at a gestational age of 26 weeks 0 days–33 weeks 6 days and who were identified as being at risk of imminent preterm birth were eligible for inclusion and invited to participate. Imminent preterm birth was defined as planned or expected birth in the next 48 h (either provider-initiated or after preterm prelabour rupture of membranes or spontaneous labour). To be eligible, a woman's gestational age had to be informed by an obstetric ultrasound. Women were not eligible if they had clinical signs of severe infection, suspicion, or evidence of clinical chorioamnionitis, major congenital fetal anomalies, concurrent or recent use of systemic steroids, were participating in another maternal or newborn health trial, or had a contraindication to steroid use. Women were screened at the time of presentation to hospital, and randomised women and their babies were followed up until day 28 after birth, at the hospital or at home. Written informed consent was obtained from every woman prior to randomisation.

For this secondary analysis, we included randomised women (and their newborns) who received a single course of dexamethasone or placebo, had complete data for trial primary outcomes, and who had complete data on time of dexamethasone or placebo administration and time of birth. Women who received a repeat course (and their newborns) were excluded. Hence, the population for this analysis comprised 92.5% of women and 90.2% of babies who participated in the WHO ACTION-I trial.

### Randomisation and masking

In the ACTION-I Trial, site-stratified individual randomization (1:1 ratio) with balanced permuted blocks of 10 were used. Eligible, consenting women were randomly assigned to a course of IM injections of either 6 mg dexamethasone or identical placebo (normal saline) administered every 12 h, to a maximum of four doses, or until hospital discharge or birth, whichever was earlier. Women were eligible for a repeat course if they had not given birth after 7 completed days but still met the eligibility criteria (the repeat course was the same as the initial allocation).

### Outcomes

The ACTION-I trial primary outcomes were neonatal death until 28 completed days of life; stillbirth or neonatal death (any baby death); and a composite outcome for possible maternal bacterial infection (defined as maternal fever or clinically suspected or confirmed infection, for which therapeutic antibiotics were used). Secondary outcomes included maternal and newborn mortality and morbidity outcomes, and process of care outcomes.

For this secondary analysis, the outcomes of interest were neonatal death, stillbirth or neonatal death (i.e., any baby death), severe neonatal respiratory distress within 24 h, and severe neonatal respiratory distress within the first week of birth (168 h). In the ACTION-I trial, severe neonatal respiratory distress was clinically defined based on the composite of fast breathing (respiratory rate ≥70 breaths per minute); and at least one of marked nasal flaring during inspiration, expiratory grunting audible with naked ear, or severe chest in-drawing; and SpO2 less than 90% or use of supplemental oxygen.

### Statistical analysis

The exposure variables of interest for this analysis were the allocated treatments (dexamethasone and placebo). We aimed to assess how selected newborn outcomes varied by administration-to-birth interval, which was defined as the time from administration of the first dose of dexamethasone or placebo until birth. This interval was considered using both categorical and continuous variable approaches. For each outcome, we explored the administration-to-birth time interval which was associated with the greatest benefit of dexamethasone compared to placebo.

We used two different statistical approaches. First, we conducted subgroup analyses stratifying the study population by pre-defined categories of administration-to-birth intervals, and calculated the relative risks of dexamethasone compared to placebo for each newborn outcome and for each interval category. This approach had two important limitations: firstly, the categories of administration-to-birth intervals were pre-defined based on clinical practice considerations and boundaries that were set from previous research. As a result of these limitations, interactions with intervals other than those categories defined *a priori* might be concealed, and it does not take full advantage of having continuous data for the administration-to-birth interval. It also does not take into account the impact of gestational age (which naturally increases with increasing administration-to-birth interval) on newborn health outcomes – it was evident from this analysis as the relative risks varied considerably depending on the gestational age at time of first dose.

To address these issues, we used a second statistical approach. We created a prediction model based on women receiving dexamethasone or placebo for each of the newborn outcomes of interest, taking into account the administration-to-birth interval as continuous variable, as well as gestational age at first administration. Then, from the predicted risks for the dexamethasone and placebo arms, we estimated the relative risks (RRs) for each outcome by gestational age at first dose to identify the interval with the greatest benefit. We used multivariate logistic regression to create the prediction models for each outcome. First, we fitted a full quadratic polynomial model for each trial arm on time from administration-to-birth and gestational age at first dose (i.e., the model included the linear and quadratic terms for each of these continuous variables and the cross product of the two), plus covariates. The following covariates were ultimately included in the model: gestational age at first dose, whether the preterm birth was spontaneously- or provider-initiated, mode of birth, maternal age, singleton or multiple birth, nulliparity, history of preterm birth, having at least one obstetrical condition present, and whether tocolytics and magnesium sulfate for fetal neuroprotection had been administered. These covariates were all measured at baseline, except for mode of birth.

We used the Lasso strategy for selecting variables^14^ and assessed the goodness of fit of several candidate models, using the difference between the log-likelihood of the saturated model and that of the fitted model. For each outcome a reduced model was obtained, keeping only terms that were significant at 10% (See Supplementary File S1 for statistical methods on model development). We assessed whether the added covariates altered the findings on which time interval had the greatest benefits of dexamethasone compared to placebo, with respect to the neonatal outcomes. We also assessed models using either gestational age at first dose or gestational age at birth - the fits and estimated risks were identical. This is due to the linear relationship between these two measures (i.e., gestational age at birth is the sum of gestational age at first dose and the time from first dose to birth).

Using the reduced model, we estimated for each gestational age at first dose the effects of dexamethasone by different administration-to-birth intervals in days up to 28 days from the start of treatment, in order to identify the time interval with the greatest benefits of dexamethasone compared to placebo. Effects of dexamethasone were expressed as relative risks (risks of outcome for infants exposed to dexamethasone compared with those exposed to placebo, with their 95% confidence intervals). To estimate the relative risks with their 95% confidence intervals, we used the Poisson distribution with the log link in the logistic model and then estimated linear combinations corresponding to the relative risks for different values of gestational age at first dose and administration-to-birth intervals. The same analysis was conducted with administration-to-birth intervals in hours up to 24 h from the start of treatment to further elucidate the relative risks of neonatal death at shorter intervals. For each gestational age at first dose, we plotted the relative risks by administration-to-birth intervals, and identified the optimal interval as the time points where greatest impact of dexamethasone (in terms of risk reduction compared to placebo) were observed.

To aid interpretation and develop a tool for potential clinical application of these findings in our study context, we used profiler software (JMP Statistical Discovery Software, version 16.0, Supplementary File S2) to generate and visualize the predicted risk of an outcome for any combination of the independent variables. The profiler shows the estimated risk and its confidence intervals for particular combinations of gestational age at first dose, administration-to-birth interval, and treatment with dexamethasone or no treatment (i.e., placebo).

### Role of the funding source

The funders of the study had no role in study design, data collection, data analysis, data interpretation, or writing of the report. All investigators had access to dataset, and OTO and RB had final responsibility for submitting for publication.

## Results

**Fig. 1:**
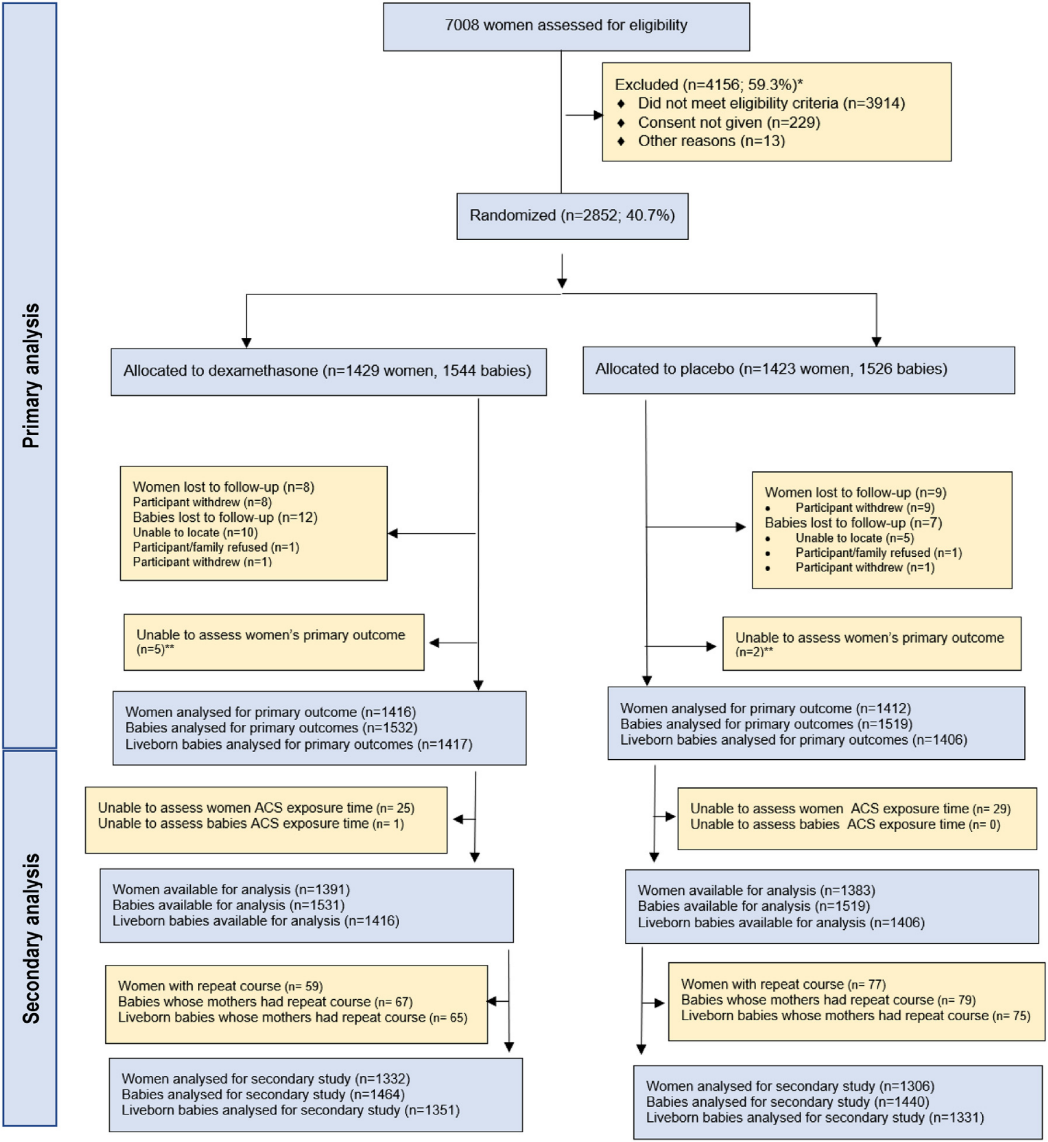
**Study flowchart.**

The study population comprised 1332 women and their 1464 babies in the dexamethasone arm and 1306 women and their 1440 babies in the placebo arm (Fig. 1). The baseline characteristics of populations of women in the dexamethasone and placebo arms were similar (Table 1). Nearly 40% of women underwent clinically indicated induction of labour or caesarean birth. The mean gestational age at recruitment was 30.9 weeks. In total, 2012/2638 (76.3%) of women gave birth before 34 weeks gestation and 2370/2638 (89.8%) gave birth during the preterm period (i.e., before 37 weeks 0 days’ gestation).

**Table 1:** Characteristics of analysis population.

Characteristic	Dexamethasone N = 1332 women	Placebo N = 1306 women
Clinical assessment of imminent preterm birth at trial entry – no. (%)		
Spontaneously-initiated preterm birth	823 (61.8)	794 (60.8)
Preterm prelabour rupture of membranes	418 (31.4)	355 (27.2)
Spontaneous preterm labour	405 (30.4)	439 (33.6)
Provider-initiated preterm birth	509 (38.2)	512 (39.2)
Gestational age at trial entry – no. (%)		
26 weeks 0 days–27 weeks 6 days	117 (8.8)	94 (7.2)
28 weeks 0 days–31 weeks 6 days	586 (44.0)	603 (46.2)
32 weeks 0 days–33 weeks 6 days	629 (47.2)	607 (46.5)
34 weeks 0 days^a^	0 (0.0)	2 (0.2)
Gestational age at trial entry (wks) – mean (SD)	30.9 (2.0)	30.9 (1.9)
Maternal age (yrs) – mean (SD)	27.3 (5.8)	27.4 (5.9)
Missing – n (%)	1 (0.1)	0 (0.0)
Educational level completed – no. (%)		
No education	166 (12.5)	158 (12.1)
Primary education only	353 (26.5)	382 (29.2)
Secondary education only	512 (38.4)	462 (35.4)
Post-secondary/tertiary education	298 (22.4)	299 (22.9)
No answer	3 (0.2)	5 (0.4)
Marital status – no. (%)		
Married/Cohabiting	1285 (96.5)	1260 (96.5)
Single/Separated/Widowed/Divorced	47 (3.5)	46 (3.5)
No. of fetuses in the current pregnancy – no. (%)		
Single	1207 (90.6)	1184 (90.7)
Twin	117 (8.8)	119 (9.1)
Higher order multiples	8 (0.6)	3 (0.2)
Parity		
0	501 (37.6)	511 (39.1)
1–2	596 (44.7)	572 (43.8)
3–4	199 (14.9)	179 (13.7)
5 or more	36 (2.7)	44 (3.4)
History of preterm birth among parous women – no. (%)		
No	642/831 (77.3)	610/795 (76.7)
Yes	163/831 (19.6)	167/795 (21.0)
Unknown	26/831 (3.1)	18/795 (2.3)
Medical conditions currently present – no. (%)^a^		
Chronic hypertension	59 (4.4)	68 (5.2)
Diabetes mellitus (non-gestational)	12 (0.9)	13 (1.0)
HIV or AIDS	30 (2.3)	27 (2.1)
Tuberculosis	1 (0.1)	2 (0.2)
Pyelonephritis	4 (0.3)	10 (0.8)
Anaemia (Hct ≤ 26% or Hb ≤ 9 g/dL)	97 (7.3)	122 (9.3)
Malaria	45 (3.4)	47 (3.6)
Obstetric conditions currently present – no. (%)^a^		
Gestational diabetes	21 (1.6)	10 (0.8)
Pre-eclampsia or eclampsia	257 (19.3)	299 (22.9)
Gestational hypertension (excl. preeclampsia or eclampsia)	70 (5.3)	60 (4.6)
Oligohydramnios (known or suspected)	326 (24.5)	292 (22.4)
Polyhydramnios (known or suspected)	17 (1.3)	30 (2.3)
Intrauterine growth restriction (known or suspected)	91 (6.8)	91 (7.0)
Macrosomia	4 (0.3)	3 (0.2)
Abruptio placentae	47 (3.5)	39 (3.0)
Placenta praevia	96 (7.2)	102 (7.8)
Other obstetric haemorrhage	62 (4.7)	40 (3.1)
Trimester of pregnancy when ultrasound for gestational age estimate was performed – no. (%)		
1st trimester (up to 13 weeks 6 days)	155 (11.6)	140 (10.7)
2nd trimester (14 weeks 0 days–27 weeks 6 days)	311 (23.3)	298 (22.8)
3rd trimester (28 weeks 0 days and beyond)	866 (65.0)	868 (66.5)
Medication administered prior to randomization – no. (%)		
Tocolytic	232 (17.4)	238 (18.2)
Magnesium sulfate for neuroprotection	126 (9.5)	157 (12.0)
Mode of birth – no. (%)		
Missing	45 (3.4)	30 (2.3)
Cephalic vaginal delivery	606 (45.5)	583 (44.6)
Breech vaginal delivery	56 (4.2)	50 (3.8)
Vacuum or forceps delivery	0 (0.0)	2 (0.2)
Caesarean section before labour onset	465 (34.9)	465 (35.6)
Caesarean section after labour onset	160 (12.0)	176 (13.5)

a 2 women were randomised at 34 weeks 0 days.

Supplementary Fig. S1 shows the distribution of the administration-to-birth interval by trial arm. For women receiving dexamethasone, 20.1% (294/1464) had an administration-to-birth interval of ≤6 h, 36.1% (529/1464) had an interval of 24 h–168 h (1 week), and 27.0% (395/1464) had an interval greater than 1 week. When considered as a continuous variable, the distribution of this interval for dexamethasone and placebo arms was very similar, though the placebo group had a slightly shorter administration-to-birth time. Supplementary Fig. S2 shows the number of births and neonatal deaths for each week of gestational age at birth - the highest number of births occurred at 33 weeks (571 babies). Across the preterm period (<37 weeks), the neonatal mortality rate was highest for babies born at 26 weeks (27/35, 77.1%) and lowest for babies born at 36 weeks (2/93, 2.2%). In the analysis population, neonatal death was 20.4% (275/1351) in the dexamethasone arm and 23.8% (317/1331) in the placebo arm, while severe respiratory distress within 168 h was 9.7% (114/1171) and 11.6% (134/1152), respectively.

**Fig. 2:**
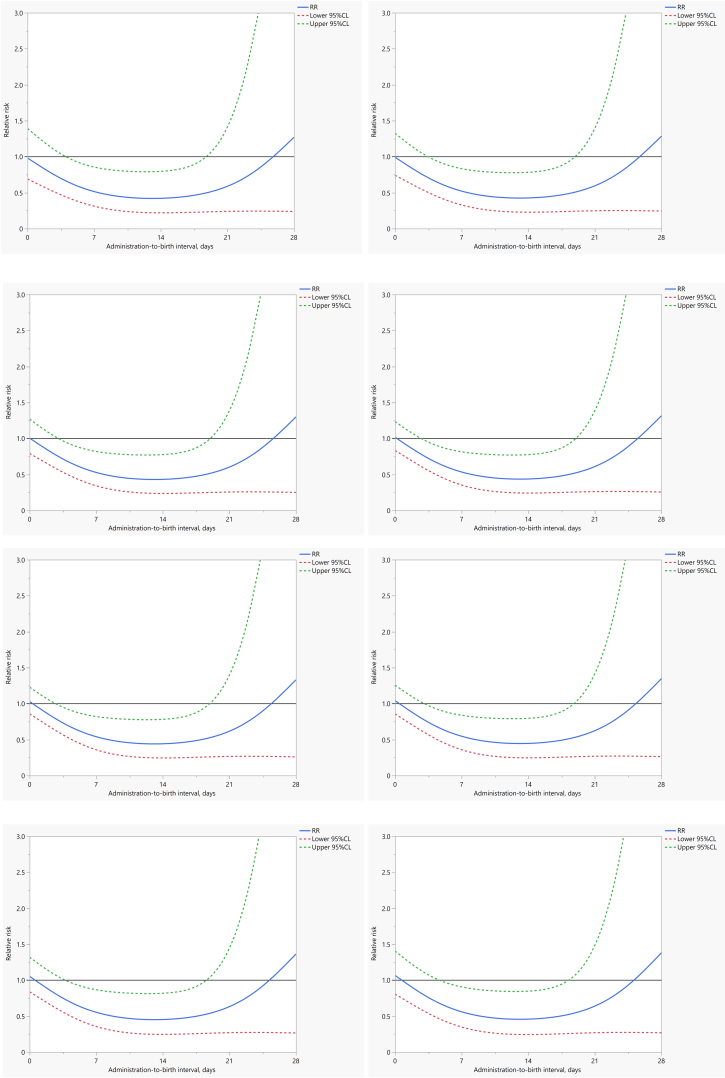
**Relative risks of neonatal mortality in preterm infants exposed to dexamethasone compared to placebo, by administration-to-birth intervals (from 0 through 28 days),at different gestational ages of first administration.**

Table 2 presents relative risks for the newborn outcomes for categories of administration-to-birth interval. While these have not accounted for gestational age at the time of first dose, there is an overall pattern of reduction in the point estimate of the relative risk of each outcome as the administration-to-birth interval increases. Fig. 2 and Supplementary Table S1a present the relative risks and 95% confidence intervals of neonatal death in preterm babies exposed to dexamethasone compared to placebo, by administration-to-birth intervals as a continuous variable (from 0 through 28 days) for different gestational ages at first dose (from 26 weeks 0 days through 33 weeks 0 days). Across all gestational ages at first dose, the point estimates for the relative risks were consistently reduced with increasing time from administration-to-birth until days 13 and 14 (i.e., nadir of the curves where the greatest risk reductions were observed), but gradually diminished thereafter and with reversed effects as it approached an administration-to-birth interval of day 28 days. Supplementary Fig. S3 and Supplementary Table S1b shows the relative risks for neonatal death by administration-to-birth interval for the first 24 h. For all gestational ages at first dose, the relative risks were not indicative of any clinical benefit or suggestive of harm throughout the 24 h following administration.

**Table 2:** Relative risks and 95% CI of neonatal outcomes comparing dexamethasone with placebo administration, per administration-to-birth interval.

Administration-to-birth interval	Neonatal death (to 28 days)	Stillbirth or neonatal death (any baby death)	Severe respiratory distress at 24 h	Severe respiratory distress at 168 h
0–6 h	1.08 (0.83–1.41)	1.11 (0.87–1.41)	0.98 (0.39–2.49)	1.10 (0.70–1.73)
>6–12 h	0.89 (0.63–1.28)	1.00 (0.72–1.40)	0.59 (0.15–2.28)	1.08 (0.61–1.92)
>12–24 h	1.02 (0.67–1.56)	1.03 (0.72–1.48)	0.92 (0.34–2.47)	0.90 (0.45–1.79)
>24 h–1 week	0.77 (0.60–0.98)	0.83 (0.68–1.00)	0.40 (0.20–0.81)	0.66 (0.44–0.98)
>1 week	0.65 (0.39–1.08)	0.67 (0.47–0.94)	0.39 (0.10–1.46)	0.81 (0.36–1.83)

Supplementary Table S1c–e show the relative risks by administration-to-birth interval for other outcomes of interest. Supplementary Fig. S4 and Supplementary Table S1c show the relationship between reduction in the risks of stillbirth or neonatal death (any baby death) and administration-to-birth interval was similar to that of neonatal death although with the nadirs occurring at days 11 and 12. For severe neonatal respiratory distress outcomes, although the overall pattern of risk reduction as administration-to-birth interval increased towards the second week was demonstrated, the precision of the data was very low and less reliable beyond that point (Supplementary Table S1d and e and Supplementary Figs. S5 and S6).

**Fig. 3:**
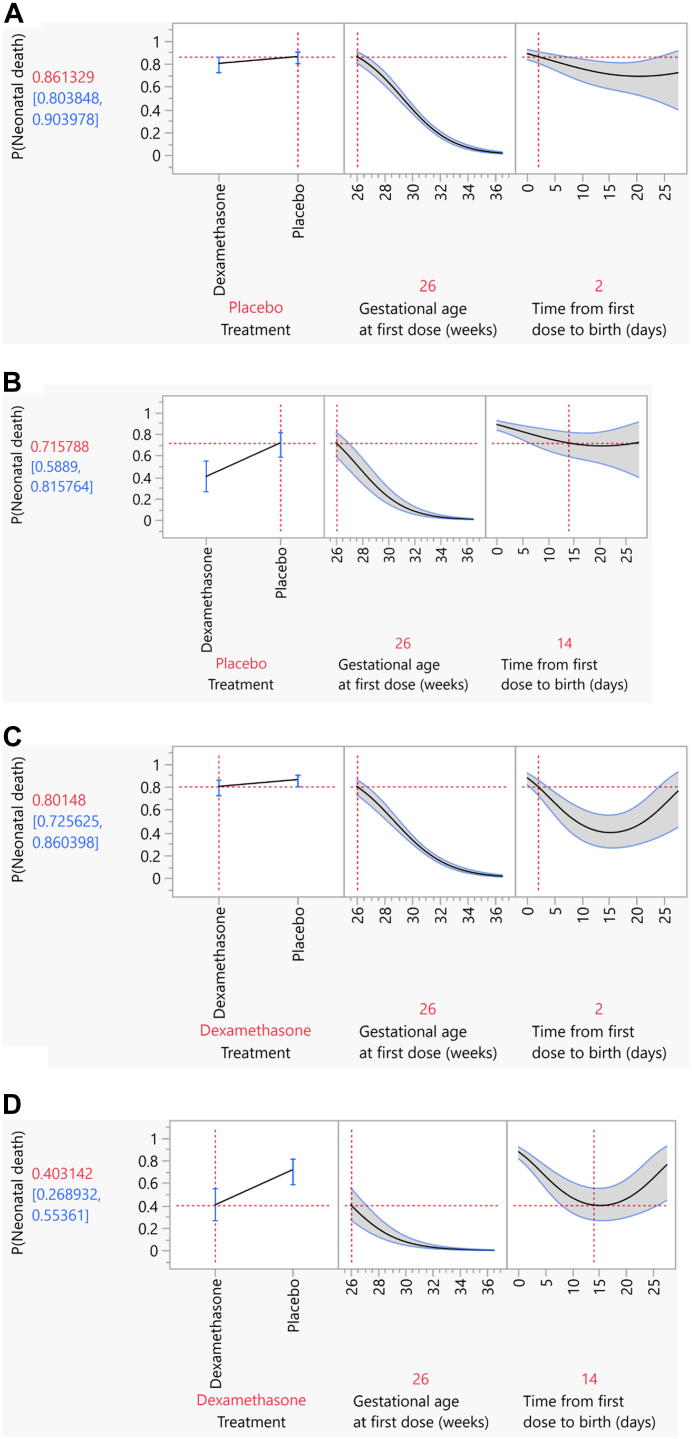
**Profiler plots of risks of neonatal mortality for infants exposed to antenatal dexamethasone and infants exposed placebo at 26 weeks 0 days gestational age at first administration.** (A) Risk of neonatal death, indicated as P(Neonatal death) with no dexamethasone treatment (i.e., placebo) initiated at 26 weeks 0 days gestation and birth occurring 48 h later, from a multivariate logistic model. (B) Risk of neonatal death, indicated as P(Neonatal death) with no dexamethasone treatment (i.e., placebo) initiated at 26 weeks 0 days gestation and birth occurring 14 days later, from a multivariate logistic model. (C) Risk of neonatal death, indicated as P(Neonatal death) with dexamethasone treatment initiated at 26 weeks 0 days gestation and birth occurring 48 h later, from a multivariate logistic model. (D) Risk of neonatal death, indicated as P(Neonatal death) with dexamethasone treatment initiated at 26 weeks 0 days gestation and birth occurring 14 days later, from a multivariate logistic model.

The profiler tool (Supplementary File S2) can be used to explore and visualise the risks of pre-specified outcomes by different administration-to-birth intervals, taking into account a woman's gestational age at time of administration and whether the woman was treated with dexamethasone. Fig. 3 provide examples of the output of this tool for neonatal death with administration starting at 26 weeks 0 days gestation and administration-to-birth intervals of 2 days (48 h) and 14 days. Panels 3A and 3B show the risks of neonatal death for two hypothetical women who were not treated with dexamethasone at 26 weeks 0 days gestation (i.e., exposed to placebo), and birth occurred at 48 h later for one woman and 14 days later for other woman, respectively. For these two scenarios, the modest difference in the risks of neonatal death for the infants born 48 h (2 days) and 14 days from first treatment dose (0.86 versus 0.72, respectively) is attributable to increasing fetal maturity due to increasing gestational age. Panels 3C and 3D show the risks of neonatal death for similar women, but who were treated with antenatal dexamethasone. The difference in neonatal death risks between 48 h (2 days) and 14 days (0.80 versus 0.40, respectively) is more substantial, and attributable to the effects of dexamethasone in addition to increasing fetal maturity as a result of increasing gestational age.

## Discussion

This study confirms that there is a strong relationship between the time from antenatal dexamethasone administration-to-birth and critical newborn outcomes. Compared to infants exposed to placebo, we found that neonatal mortality risk further reduced with increasing administration-to-birth interval for infants exposed to a single course of dexamethasone between 26 weeks 0 days and 33 weeks 6 days. While gestational age is a well-known critical mediator of preterm newborn outcomes, the observed relationship was independent of the effects of natural fetal maturation alone. Peak mortality reduction was observed by days 13 and 14, and then diminishing as the interval approached 28 days, irrespective of the gestational age at the time of first treatment dose. The impact of dexamethasone on neonatal mortality reduction during the first 24 h following initiation of treatment is uncertain, although the overall trend is in favour of risk reduction as the interval increases. The observation for clinically assessed severe respiratory distress outcomes is less marked, although the overall pattern of risk reduction as the interval extends into the second week is consistent with that of neonatal death.

While these findings of a strong relationship between dexamethasone administration-to-birth interval and newborn survival is conceptually consistent with previous studies in terms of a beneficial window, it also challenges the generally held view of 24 h to 7 days as the “optimal interval”, and what has been otherwise described as sub-optimal or potentially harmful intervals. Our study suggests that the beneficial window may start sooner than the 24 h threshold and extends through to the end of second week following administration, even in the absence of a second course of ACS treatment.

We conducted a systematic review of randomised and non-randomised studies on the association between different antenatal corticosteroid administration-to-birth intervals and maternal and newborn outcomes (14). The review identified 59 studies (11 trials and 48 observational studies), 39 of which were conducted in high-income country settings. One of the 11 trials was the WHO ACTION-I trial primary analysis, and none of the other 10 trials evaluated ACS administration-to-birth interval as a continuous measure, nor adjusted for the confounding effect of gestational age at time of administration. Findings in the current analysis have not been reported previously in efficacy trials. Three trials reported on neonatal mortality for different categories of ACS administration-to-birth interval - all three were small (ranging from 73 to 208 newborns) and reported no differences in neonatal death across intervals (15–17). Seven trials (with 80–315 newborns) reported that odds of RDS were not different for any ACS administration-to-birth interval (15, 17–22). However, one trial (696 newborns) found reduced odds of RDS at 1–7 days and >7 days with ACS compared to no ACS treatment (23). One other trial (73 newborns) found reduced odds of RDS at ≥8 days compared to no ACS treatment (24).

Large observational studies from Canada and Australia, as well as multi-country studies from other high-resource settings, have explored the relationship between the ACS administration-to-birth interval and newborn outcomes.^7^^,^^8^^,^^15^^,^^16^ Comparing findings across these studies is challenging given the differences in enrolled populations, variations in the categories of time intervals used, and use of different ACS regimens. Melamed et al.^7^ analysed ACS effectiveness in a Canadian retrospective cohort of 6870 infants born at 24 to <34 weeks, and reported that ACS (usually betamethasone or possibly dexamethasone) had maximum benefit at 1–7 days, while at <24 h or >7 days the odds of neonatal mortality and morbidity were relatively poorer. Conversely, Norman et al.^8^ analysed 4594 singletons born at 24–31 weeks across multiple European countries - 78.9% of obstetric units were using betamethasone, 4.9% used dexamethasone and 15.4% used either. They observed significant declines in neonatal mortality at <24 h associated with ACS use. While the greatest reductions in neonatal mortality, severe neonatal morbidity and severe neonatal brain injury were between 24 h and 7 days, the mortality benefits appeared to persist beyond 3 weeks after administration. It is important to note that while these studies suggested that 24 h to 7 days had maximal benefits compared to shorter and longer intervals, they also confirmed that any category of ACS administration-to-birth interval was better than no ACS treatment. The interpretation, therefore, is that the 24 h and 7 days thresholds are arbitrary and not necessarily inflection points outside which ACS is not beneficial or associated with harms. The initial exploration of relative risks of newborn outcomes by categories (Table 2) shows that infants born from >24 h to 1 week after treatment was the only category with point estimates and 95% confidence intervals suggestive of significant benefits for all specified outcomes. This could have led to a similarly erroneous conclusion of benefit being confined to this interval, if subjected to the same type of analysis as previous studies.

The strengths of this study include the use of data from a multi-country, randomised, placebo-controlled, double-blind trial, in which the administration-to-birth interval was prospectively reported with high precision, and newborn health outcomes were measured robustly and systematically, and there were high event rates for newborn primary outcomes. This secondary analysis included 92% of women and 95% of babies who participated in the original trial. This secondary analysis was anticipated during the design phase of the trial and thus allowed for a proactive collection of accurate data on time of treatment and time of birth. Multiple analytical approaches were used to explore associations between administration-to-birth interval and newborn outcomes. The robustness of the data set for neonatal mortality outcome allowed for construction of a tool that could potentially be applied in the clinical context of the countries where WHO ACTION-I trial was conducted. Nonetheless, we acknowledge several limitations, including that this was a secondary analysis restricted to trial participants who received a single course of dexamethasone or placebo. The analysis relied on a post-randomisation exposure variable (i.e., time from dexamethasone administration to birth) and is therefore prone to confounders which were not systematically captured. The relationships between fetal maturation, time and dexamethasone are complex, and residual confounding, or other unknown factors, may be playing a role. We have a limitation in drawing reliable inferences for the two clinically assessed severe respiratory distress outcomes, as the risk and relative risk estimates for these outcomes have low precision. This might be in part explained by the lower prevalence recorded for these secondary outcomes in the trial population, probably as a result of some degree of under-recognition of newborn respiratory complications based on clinical assessments alone (3). This is in contrast to the prevalence of neonatal mortality, which was the primary outcome upon which the trial sample size estimation was based. There were also fewer observations with longer administration-to-birth intervals, leading to greater uncertainty (wider confidence intervals). A number of other factors related to the analysis population might affect generalizability of these findings, such as the enrolment of women at risk of imminent preterm birth from 26 weeks 0 days–33 weeks 6 days only; that two-thirds of women had a gestational age estimate based on third trimester ultrasound; and that the ACS regimen was IM dexamethasone phosphate. Further validation of these findings in other, high-quality datasets would be useful.

Our study confirms that the window of benefit in terms of neonatal mortality reduction following dexamethasone administration is time bound, but the inflection point and subsequent diminution do not occur until about two weeks following the initiation of treatment. In view of the challenges of accurately predicting the timing of spontaneous preterm birth, this longer optimal administration-to-birth interval provides reassurance regarding the benefits of ACS in situations where anticipated birth occurs after the conventional 7 days’ window. As the mortality risk among infants who were exposed to a single course of dexamethasone is lower for the larger part of 28 days following treatment, administration of a repeat (or “rescue”) course during this period to optimise the administration-to-birth interval might be unnecessary. However, this warrants further investigation, as trials of repeat ACS regimens have thus far used betamethasone only (27).

From a clinical standpoint, clinicians may consider prolonging the time of birth with tocolytics where it is deemed clinically safe, not just for ensuring that all prescribed doses of ACS are administered, but also to optimise the administration-to-birth interval, while continually weighing the balance of benefits of increasing interval against risk of fetal exposure to an adverse intrauterine environment. The tool developed from WHO ACTION-I data could be applied in hospitals in low-resource countries with similar characteristics as in the trial to estimate risks of dexamethasone treatment versus no treatment, in situations where the administration-to-birth interval can be pre-determined with a high degree of certainty, such as in provider-initiated preterm birth. The tool could also be used for support clinical assessment and estimation of the risks of adverse preterm newborn outcomes at the time of birth when all variables, including the administration-to-birth interval, are known.

Our findings call for a review of the 2015 WHO recommendation that suggests that antenatal corticosteroid should only be applied when preterm birth is considered “imminent” within 7 days of starting treatment, which was based on subgroup analysis that is no longer supported in the current updates of Cochrane review underpinning the recommendation.^1^ Although the current study does not refute the suggestion that there may be benefit when ACS is administered in situations where birth is anticipated within 24 h, such practice should be the exception rather than the norm, as it is clear from these findings that a minimum number of days following ACS administration is required to maximise benefits and avoid harms.

Further investigation is required on the overarching question of what administration-to-birth interval confers the best clinical outcomes for preterm newborns. We acknowledge that it is methodologically and ethically impossible to randomise women to different administration-to-birth intervals. Therefore, combining existing WHO ACTION-I trial data with similar high-quality data sets on administration-to-birth could be the starting point to strengthen available evidence. Any future studies on this question, including observational studies, should analyse prospectively documented administration-to-birth interval as a continuous measure, and avoid arbitrary cut-offs that tend to hide the nuances in the risks of adverse outcomes within administration-to-birth interval categories. Future research could also benefit from individual patient data meta-analysis to produce more generalisable information and understand whether the observed patterns differ by population subgroups of women at risk of preterm birth. The application of machine learning to large observational and randomised trial data sets to improve prediction of the timing of preterm birth, and individualise risk estimation by time of administration-to-birth interval is long overdue.

In conclusion, this secondary analysis of a multi-country randomised trial indicates that the neonatal mortality reduction benefits from antenatal dexamethasone for women at risk of preterm birth prior to 34 weeks’ gestation may be substantially higher with longer administration-to-birth intervals than previously thought. Risk reduction for important neonatal outcomes tend to continue to increase into the latter part of second week from the start of dexamethasone administration, regardless of gestational age at time of administration.

## Contributors

The concept for this secondary analysis was conceived by O.T.O. and J.P.V., which was developed with G.P., J.C., and A.D.C. Statistical analysis was performed by G.P. and J.C., with findings presented and reviewed by all authors. J.P.V. and O.T.O. wrote the first drafts of the article, with input from F.A., G.P., R.B., S.P.N.R. and A.D.C., which was then reviewed and revised by all named authors. G.P. and J.C. have accessed and verified the underlying data.

## Data sharing statement

Request for access to these data can be made to the World Health Organization through srhmph@who.int. Data sharing with any individual or organization will be subject to WHO data sharing policy.

## Declaration of interests

All authors declare no competing interests.

## Appendix A Supplementary data

Appendix

CONSORT Checklist

Caption for Supplementary Material

Profiler of P_spl_Baby death_spl_.htm

Profiler of P_spl_Neonatal Death_spl_.htm

Profiler of P_spl_srd24h_spl_.htm

Profiler of P_spl_SRDwithin168h_spl_.htm
