# Supplementary material for: Effect of dexamethasone on newborn survival at different administration-to-birth intervals: A secondary analysis of the WHO ACTION (Antenatal CorticosTeroids for Improving Outcomes in Preterm Newborn)-I trial
Source: eClinicalMedicine. 2022 Nov 14;53:101744. doi: 10.1016/j.eclinm.2022.101744 (PMC9716334; doi:10.1016/j.eclinm.2022.101744)
Supplement: Appendix [file mmc1.pdf]

Supplementary File S1. Statistical methods for model development

Model development

The effect of treatment and administration-to-birth interval on the probability of each outcome was assessed using logistic models.

Models

Model 1

For subgroup analysis of each outcome by administration-to-birth interval we used a logistic model containing terms for treatment (*treat*), centre (*centre*) and administration-to-birth interval (*timeint*, consisting of 5 intervals or categories):

$$y = \log\left(\frac{p}{1-p}\right) = \mu + treat + centre + timeint$$

where
y=logit for binary neonatal outcome
p=proportion of events for binary neonatal outcome,

$$p = \frac{1}{1+e^{-y}}$$

Model 2

The results from Model 1 for each administration-to-birth interval did not account for the fact that the gestational ages at birth may be different for different time intervals. To account for this, either gestational age at birth or gestational age at first dose should be considered. We elected to consider both administration-to-birth time and gestational age as continuous variables, as categorizing continuous variables results in loss of information.

We fitted the following model (Model 2) and examined the significance of each term of a full second-degree polynomial:

$$y = \log\left(\frac{p}{1-p}\right) = \mu + treat + time(treat) + ga(treat) + time * time(treat) + ga * ga(treat) + time * ga(treat)$$

where
y=logit for binary neonatal outcome
p=proportion of events for binary neonatal outcome
treat=treatment
time= time from first dose to birth, in hours
ga=gestational age at first dose, in weeks

The notation A(B) means factor A nested within factor B.

We examined the results of the fitting for each outcome and kept only terms that were significant at 10%, with the proviso that if the interaction of gestational age by time was significant, then terms for the full second-degree polynomial should be included (linear and quadratic for both GA and time and the product of the linear terms). The final models are shown in the following table:

Outcome	Terms included in Model 2 for ga and time
Neonatal death	ga linear, time linear and quadratic
Any baby death	ga linear, time linear and quadratic
Severe respiratory distress at 24 hours	Full polynomial
Severe respiratory distress within 168 hours	ga linear, time linear and quadratic

For neonatal death, for example, the following table shows the results for the fitting, supporting the choice of the model "ga linear, time linear and quadratic" for the neonatal death outcome (note that the terms for ga quadratic and the product are clearly not significant):

Source	DF	Wald ChiSquare	Prob > ChiSquare
Gestational age at first dose (weeks)[Treatment]	2	130.79186	<.0001*
Time from first dose to birth (days)[Treatment]	2	70.793579	<.0001*
Treatment	1	8.4863371	0.0036*
Time from first dose to birth (days)*Time from first dose to birth (days)[Treatment]	2	11.165696	0.0038*
Gestational age at first dose (weeks)*Gestational age at first dose (weeks)[Treatment]	2	1.8975437	0.3872
Time from first dose to birth (days)*Gestational age at first dose (weeks)[Treatment]	2	0.2826478	0.8682

We used the above model adding **number of doses** and found that number of doses and administration-to-birth time were highly correlated and only one of these two variables could be kept in the model.

We used **gestational age at birth** and gestational age at first dose and found that these two variables were interchangeable, and only one of them should be kept in the model. We used gestational age at first dose instead of gestational age at birth because the latter is confounded with the time from ACS administration until birth. The time interval between trial entry and birth is thus split into two non-overlapping time intervals (gestational age at first dose and time from first dose to birth). In the analysis, the latter is termed "administration-to-birth interval".

Goodness of fit of the models was assessed by the difference between the log-likelihood of the saturated model and that of the fitted model.

Model 3

We explored the effect of other baseline variables on the probability of occurrence of each outcome, in addition to gestational age at first dose. The following covariates were included in a full model: centre, spontaneous or provider-initiated preterm birth, mode of birth, maternal age, singleton or multiple births, parity, history of preterm birth, having at least one obstetrical condition present, administration of tocolytics and administration of magnesium sulfate. We used a model like the selected Model 2 for each outcome, with the addition of these covariates (see the tests for the effects of covariates below). We used a profiler tool to assess whether the covariates that were significant affected the optimal administration-to-birth time.

Tests of effects of covariates on outcomes from Model 3

Neonatal death

Table 1. Results from model of risk of neonatal death with covariates

Source	DF	Wald ChiSquare	Prob > ChiSquare
Gestational age at first dose (weeks)/Treatment	2	327.77147	<.0001*
Time from first dose to birth (days)/Treatment	2	35.520824	<.0001*
Centre	5	24.532867	0.0002*
Time from first dose to birth (days) Quadratic/Treatment	2	14.596365	0.0007*
Treatment	1	8.0031639	0.0047*
Tocolytic medication	1	7.7384273	0.0054*
Spontaneous or provider-initiated delivery	2	8.7785301	0.0124*
Any obstetrical condition	1	3.6155696	0.0572
Maternal age (years)	1	2.9181023	0.0876
Mode of delivery	1	2.1092231	0.1464
Magnesium sulfate	1	0.9296741	0.3349
Parity	3	2.2378972	0.5245
Single or multiple pregnancy	1	0.3512341	0.5534
Any medical condition	1	0.2621698	0.6086

Table 1 shows that gestational age at first dose (weeks) within treatment, time from first dose to birth (days) within treatment (both linear and quadratic terms), centre, treatment, tocolytic medication and spontaneous or provider-initiated delivery were significant at 5%, and any obstetrical condition and maternal age at 10%.

The covariates centre, tocolytic medication, spontaneous or provider-initiated delivery, any obstetrical condition and maternal age, although significant at 10%, did not affect meaningfully the optimal time from first dose to birth.

A separate logistic model with history of preterm birth excluding parity (because of collinearity) gave p-value=0.1074 for the effect of history of preterm birth.

Any baby death

Table 2. Results from model of risk of stillbirth or neonatal death (any baby death) with covariates

Source	DF	Wald ChiSquare	Prob > ChiSquare
Gestational age at first dose (weeks)[Treatment]	2	368.6944	<.0001*
Spontaneous or provider-initiated delivery	2	29.335385	<.0001*
Centre	5	29.459719	<.0001*
Time from first dose to birth (days)[Treatment]	2	17.10086	0.0002*
Any obstetrical condition	1	7.8408535	0.0051*
Tocolytic medication	1	5.9003204	0.0151*
Mode of delivery	1	5.4004471	0.0201*
Magnesium sulfate	1	4.6865868	0.0304*
Time from first dose to birth (days)*Time from first dose to birth (days)[Treatment]	2	5.3801917	0.0679
Treatment	1	3.0089413	0.0828
Parity	3	3.8201661	0.2815
Single or multiple pregnancy	1	0.2685891	0.6043
Any medical condition	1	0.06928	0.7924
Maternal age (years)	1	0.008616	0.9260

Table 2 shows that gestational age at first dose (weeks) within treatment, time from first dose to birth (days) within treatment (both linear and quadratic), spontaneous or provider-initiated delivery, centre, any obstetrical condition, tocolytic medication, mode of delivery, magnesium sulfate and treatment were significant at 10%.

The covariates spontaneous or provider-initiated delivery, centre, any obstetrical condition, tocolytic medication, mode of delivery and magnesium sulfate, although significant at 5%, did not meaningfully affect the optimal time from first dose to birth.

A separate logistic model with history of preterm birth excluding parity (because of collinearity) gave p-value=0.4685 for the effect of history of preterm birth.

Severe respiratory distress at 24 hours

Table 3. Results from model of risk of SRD at 24 hours with covariates

**Source**	**DF**	**Wald ChiSquare**	**Prob > ChiSquare**
Gestational age at first dose (weeks)[Treatment]	2	20.02566	<.0001*
Gestational age at first dose (weeks)*Gestational age at first dose (weeks)[Treatment]	2	6.7180833	0.0348*
Treatment	1	4.102957	0.0428*
Mode of delivery	1	2.3938162	0.1218
Centre	5	6.9850456	0.2218
Any medical condition	1	1.431127	0.2316
Magnesium sulfate	1	1.1122465	0.2916
Any obstetrical condition	1	0.6184333	0.4316
Tocolytic medication	1	0.5636841	0.4528
Maternal age (years)	1	0.3090814	0.5782
Spontaneous or provider-initiated delivery	2	1.040419	0.5944
Single or multiple pregnancy	1	0.2462098	0.6198
Time from first dose to birth (days)*Time from first dose to birth (days)[Treatment]	2	0.9351028	0.6265
Time from first dose to birth (days)*Gestational age at first dose (weeks)[Treatment]	2	0.7964828	0.6715
Parity	3	1.077796	0.7824
Time from first dose to birth (days)[Treatment]	2	0.2526245	0.8813

Table 3 shows that only gestational age at first dose (weeks) (both linear and quadratic terms) and treatment were significant at 5%.

A separate logistic model with history of preterm birth excluding parity (because of collinearity) gave p-value=0.8776 for history of preterm birth.

Severe respiratory distress within 168 hours

Table 4. Results from model of risk of SRD within 168 hours hours with covariates

Source	DF	Wald ChiSquare	Prob > ChiSquare
Gestational age at first dose (weeks)[Treatment]	2	38.353201	<.0001*
Centre	5	20.014154	0.0012*
Mode of delivery	1	7.6344477	0.0057*
Time from first dose to birth (days)[Treatment]	2	8.1837864	0.0167*
Treatment	1	1.8859677	0.1697
Magnesium sulfate	1	0.9861495	0.3207
Tocolytic medication	1	0.832084	0.3617
Any obstetrical condition	1	0.6260237	0.4288
Maternal age (years)	1	0.593842	0.4409
Spontaneous or provider-initiated delivery	2	1.3252374	0.5155
Parity	3	1.8254825	0.6094
Time from first dose to birth (days)*Time from first dose to birth (days)[Treatment]	2	0.8936125	0.6397
Single or multiple pregnancy	1	0.0779989	0.7800
Any medical condition	1	0.0293113	0.8641

Table 4 shows that gestational age at first dose (weeks), time from first dose to birth (linear term), centre and mode of delivery were significant at 5%. Centre and mode of delivery, although significant at 1%, did not affect importantly the optimal time from first dose to birth.

A separate logistic model with history of preterm birth excluding parity (because of collinearity) gave p-value=0.3045 for history of preterm birth.

Estimation of relative risks

We used a profiler tool to assess whether the covariates that were significant affected the optimal administration-to-birth time, and we found that this was not the case. Therefore, the treatment effect, in terms of relative risk with 95% confidence intervals, was calculated from Model 2 using the Poison distribution and the log link. For different values of gestational age and administration-to-birth intervals we estimated linear combinations of the parameters corresponding to the relative risks. The relative risks and confidence intervals thus obtained were plotted versus administration-to-birth intervals for each gestational age.

Supplementary Figure S1. Distribution of administration-to-birth interval by trial arm

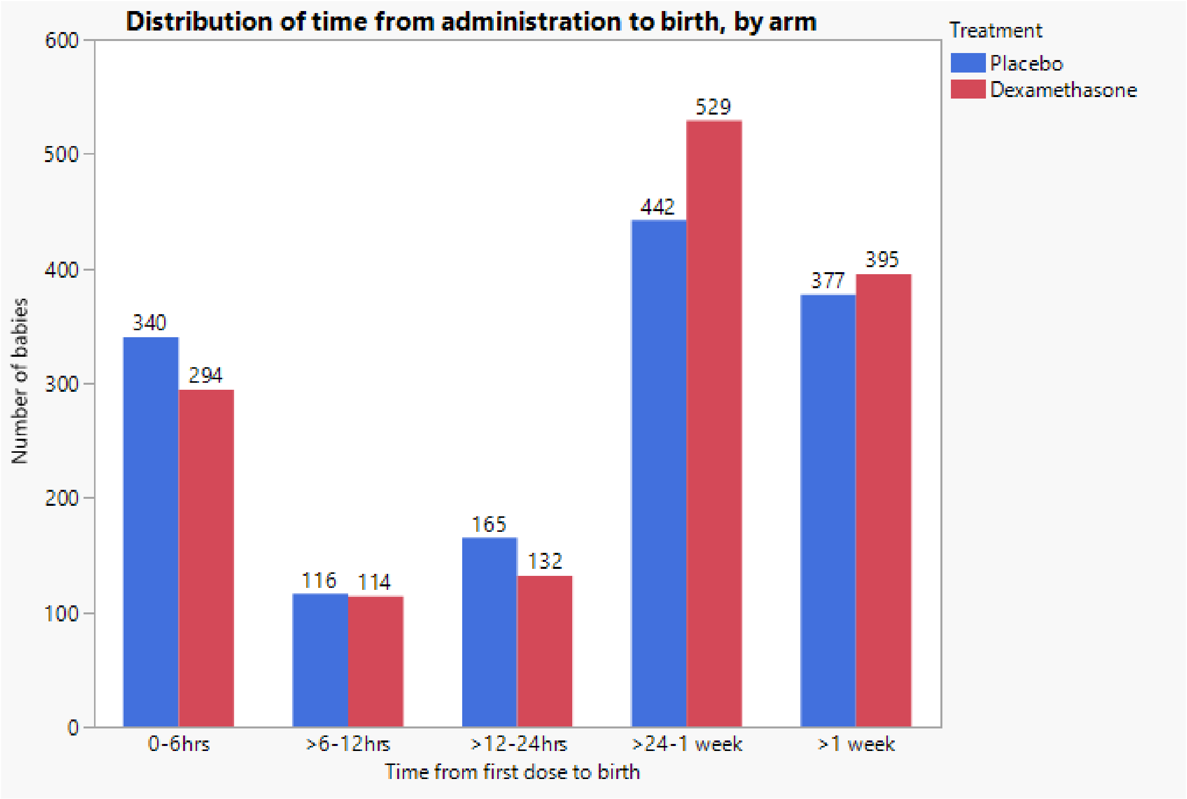

Chi-square test stratified by centre:
Cochran-Mantel-Haenszel (CMH) Chi-sq=14.75, df=4, p-value=0.0052

Supplementary Figure S2. Number of births and number of neonatal deaths by week of gestational age at birth

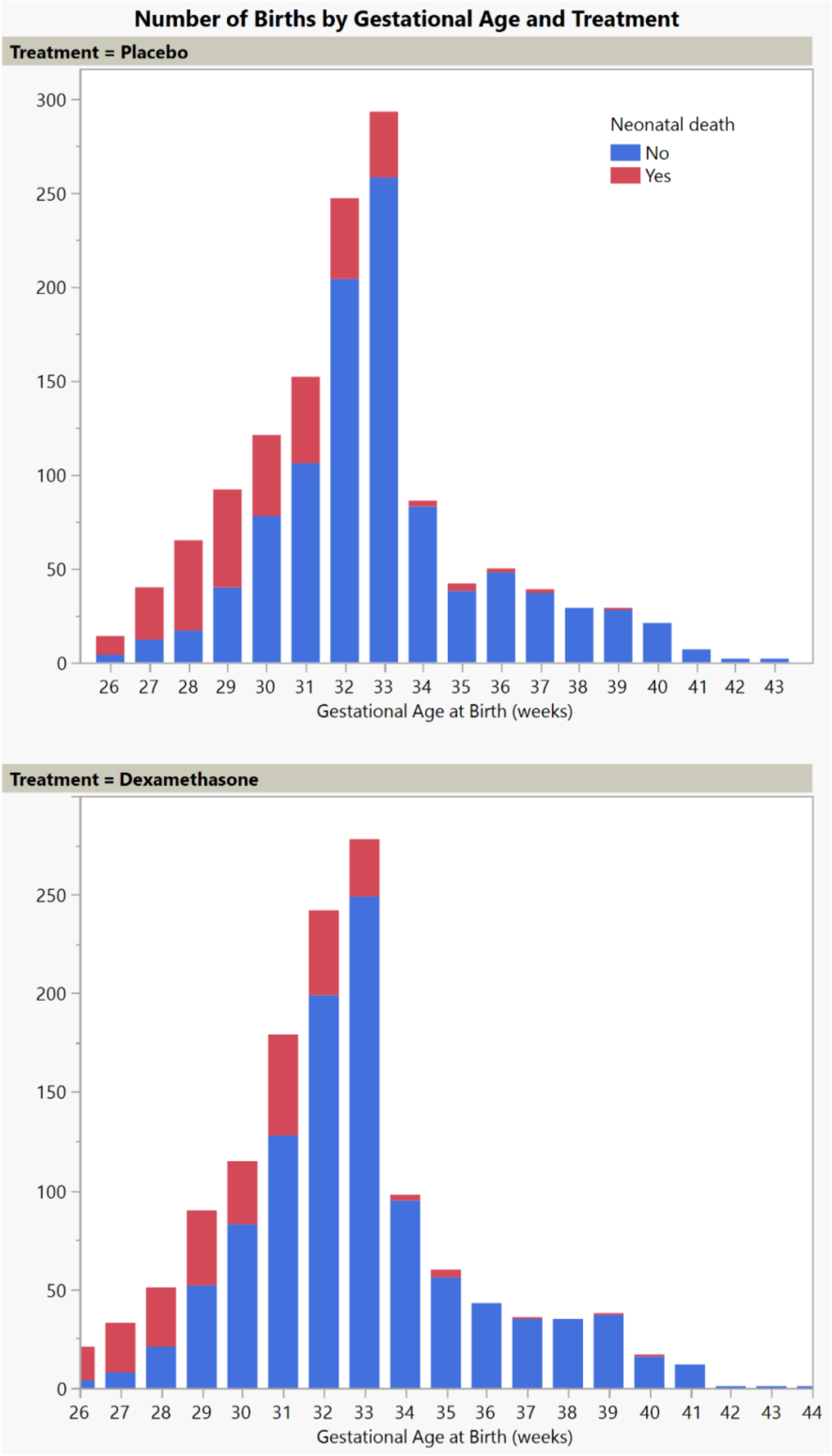

Number of Births by Gestational Age and Treatment
Treatment = Placebo
Neonatal death
No
Yes
300
250
200
150
100
50
0
26 27 28 29 30 31 32 33 34 35 36 37 38 39 40 41 42 43
Gestational Age at Birth (weeks)
Treatment = Dexamethasone
250
200
150
100
50
0
26 27 28 29 30 31 32 33 34 35 36 37 38 39 40 41 42 43 44
Gestational Age at Birth (weeks)

Supplementary Figure S3. Relative risks of neonatal mortality in preterm infants exposed to dexamethasone compared to placebo, by administration-to-birth intervals (from 0 through 24 hours), at different gestational ages at first administration

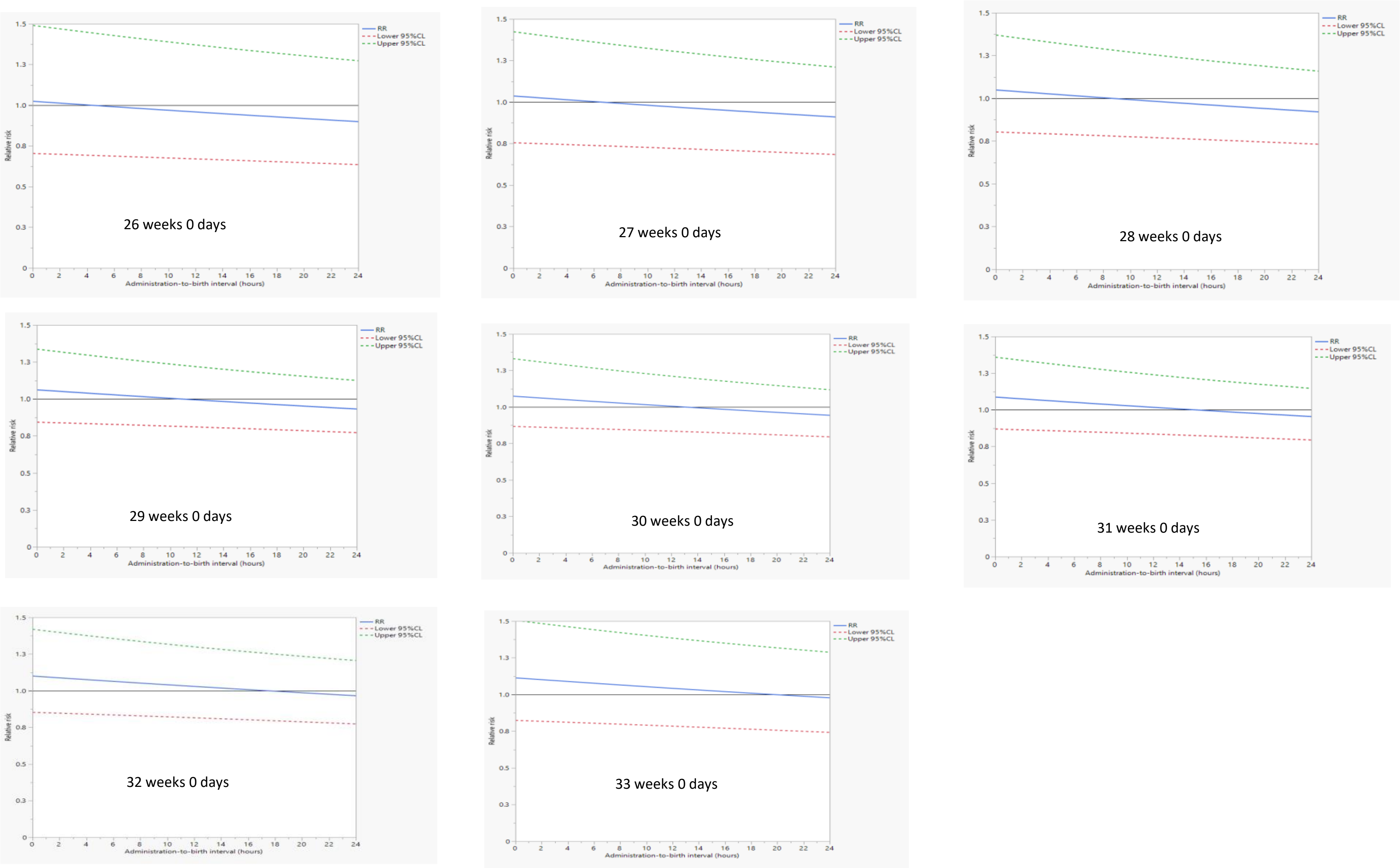

Supplementary Figure S4. Relative risks of and stillbirth or neonatal mortality (any baby death) in preterm infants exposed to dexamethasone compared to placebo, by administration-to-birth intervals (from 0 through 28 days), at different gestational ages at first administration

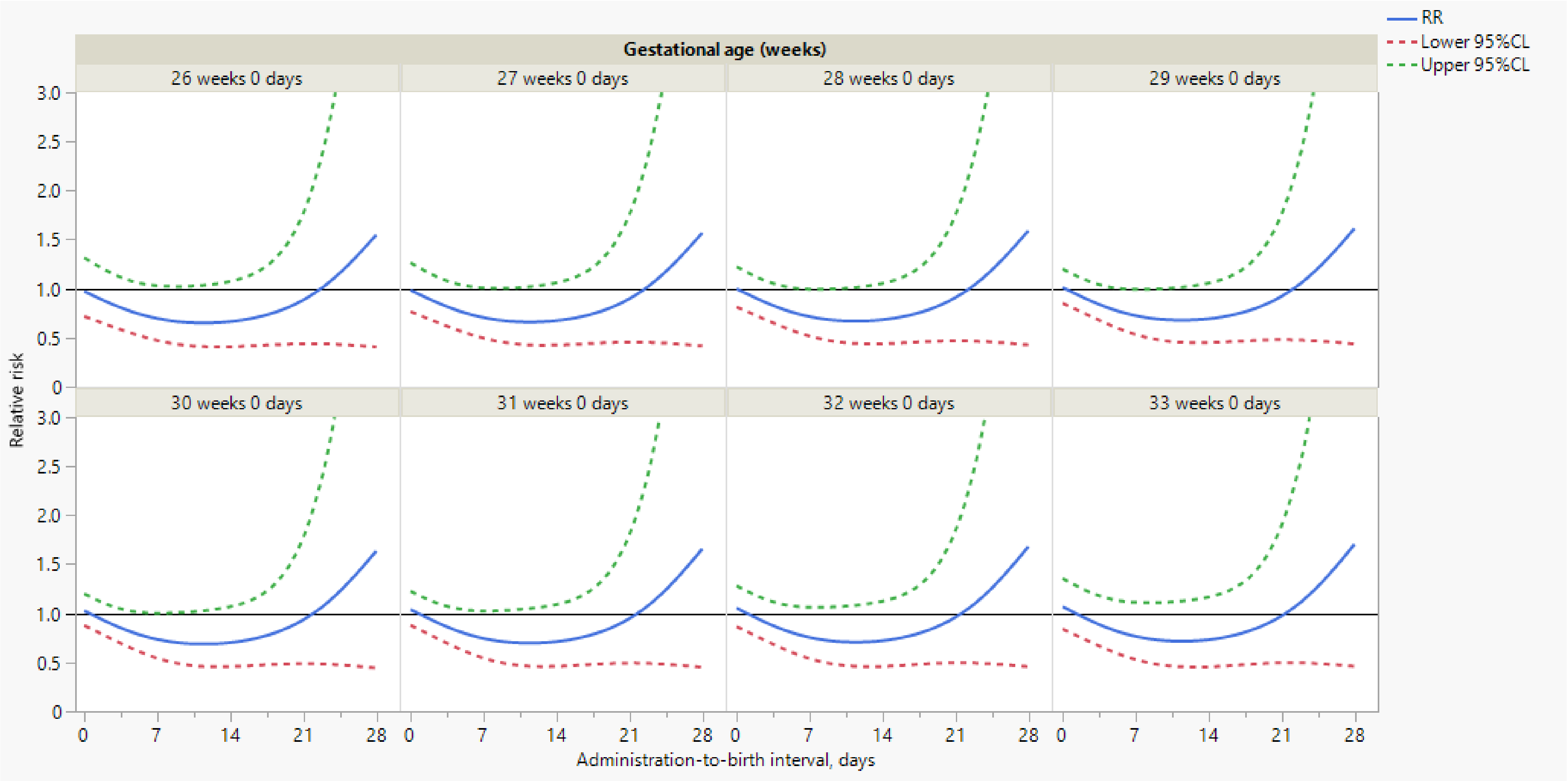

Supplementary Figure S5. Relative risks of severe respiratory distress at 24 h in preterm infants exposed to dexamethasone compared to placebo, by administration-to-birth intervals (from 0 through 28 days), at different gestational ages at first administration

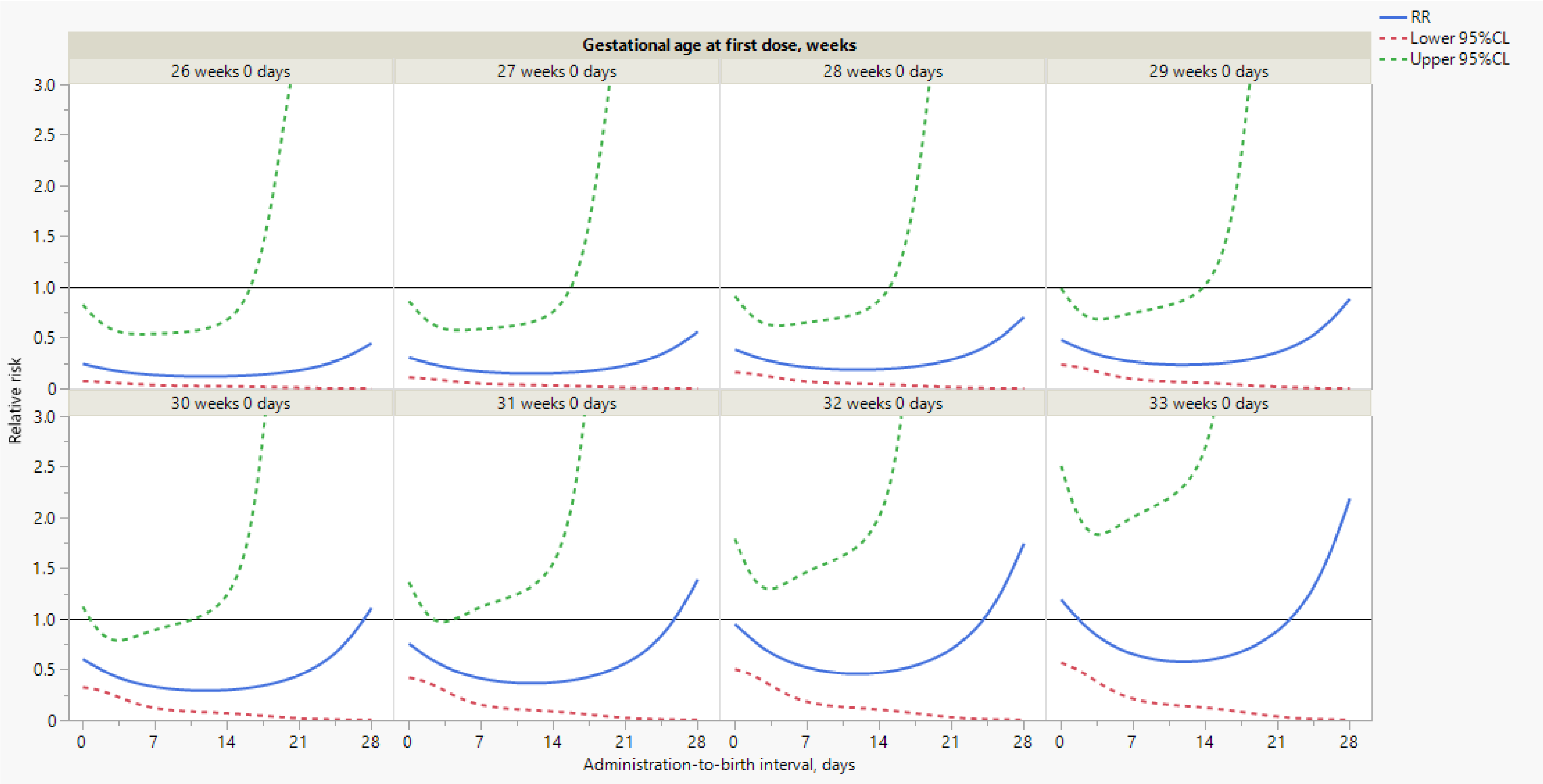

Supplementary Figure S6. Relative risks of severe respiratory distress at 168 h in preterm infants exposed to dexamethasone compared to placebo, by administration-to-birth intervals (from 0 through 28 days), at different gestational ages at first administration

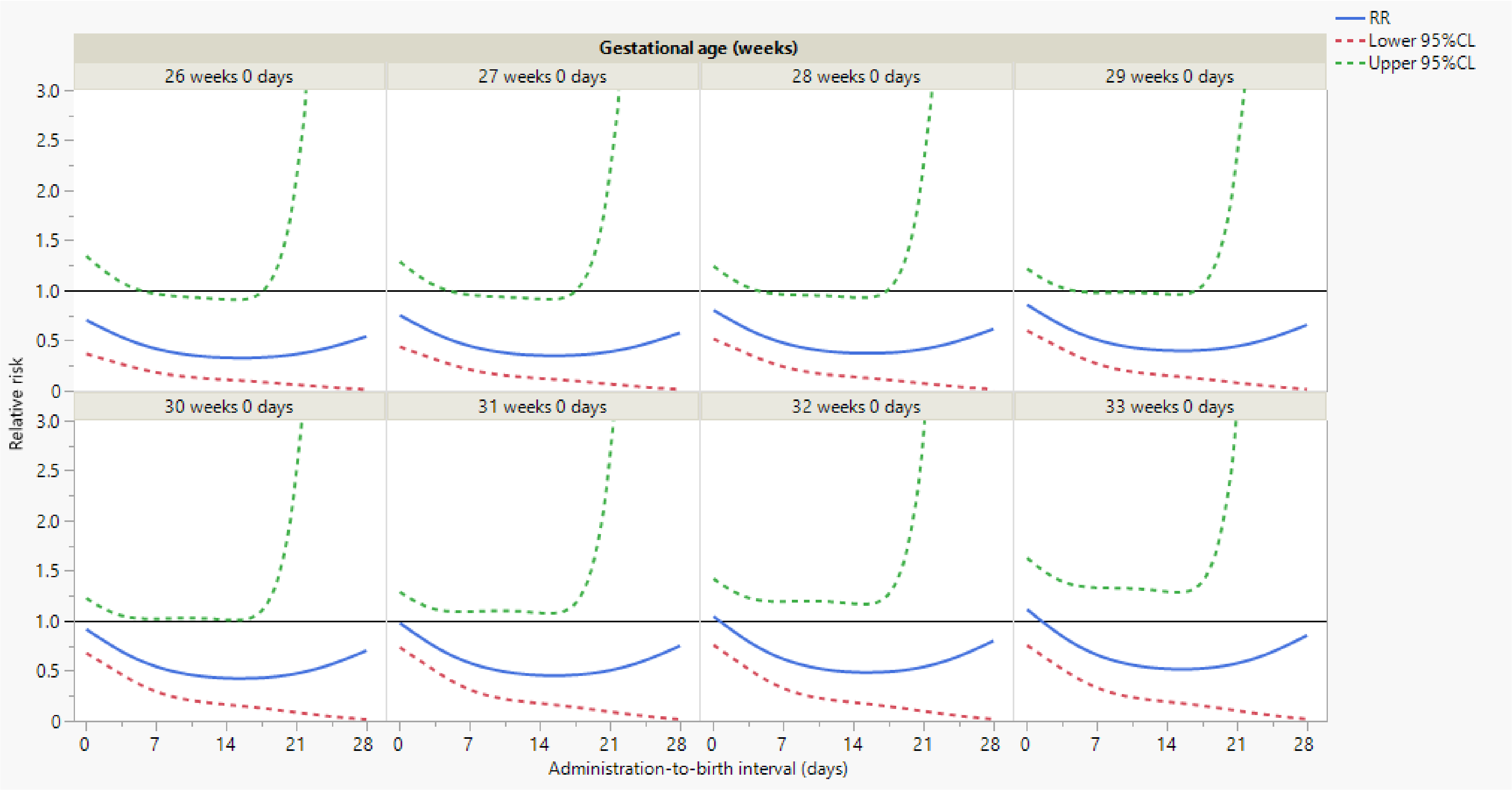

Supplementary Table 1a. Neonatal death (0-28 days)

Label	Gestational age at first dose, weeks	Administration-to-birth interval, days	RR	Lower 95%CL	Upper 95%CL
Dexa vs Placebo/ga=26 and time=0	26	0	1.02	0.70	1.49
Dexa vs Placebo/ga=26 and time=1	26	1	0.90	0.64	1.27
Dexa vs Placebo/ga=26 and time=2	26	2	0.80	0.56	1.13
Dexa vs Placebo/ga=26 and time=3	26	3	0.72	0.49	1.04
Dexa vs Placebo/ga=26 and time=4	26	4	0.65	0.43	0.97
Dexa vs Placebo/ga=26 and time=5	26	5	0.59	0.38	0.92
Dexa vs Placebo/ga=26 and time=6	26	6	0.55	0.34	0.89
Dexa vs Placebo/ga=26 and time=7	26	7	0.51	0.31	0.86
Dexa vs Placebo/ga=26 and time=8	26	8	0.48	0.28	0.84
Dexa vs Placebo/ga=26 and time=9	26	9	0.46	0.26	0.82
Dexa vs Placebo/ga=26 and time=10	26	10	0.44	0.24	0.81
Dexa vs Placebo/ga=26 and time=11	26	11	0.43	0.23	0.80
Dexa vs Placebo/ga=26 and time=12	26	12	0.43	0.23	0.80
Dexa vs Placebo/ga=26 and time=13	26	13	0.42	0.22	0.81
Dexa vs Placebo/ga=26 and time=14	26	14	0.42	0.22	0.82
Dexa vs Placebo/ga=26 and time=15	26	15	0.43	0.22	0.84
Dexa vs Placebo/ga=26 and time=16	26	16	0.44	0.22	0.88
Dexa vs Placebo/ga=26 and time=17	26	17	0.46	0.23	0.93
Dexa vs Placebo/ga=26 and time=18	26	18	0.48	0.23	1.00
Dexa vs Placebo/ga=26 and time=19	26	19	0.51	0.23	1.10
Dexa vs Placebo/ga=26 and time=20	26	20	0.54	0.24	1.23
Dexa vs Placebo/ga=26 and time=21	26	21	0.58	0.24	1.42
Dexa vs Placebo/ga=26 and time=22	26	22	0.64	0.24	1.67
Dexa vs Placebo/ga=26 and time=23	26	23	0.70	0.24	2.03
Dexa vs Placebo/ga=26 and time=24	26	24	0.78	0.24	2.51
Dexa vs Placebo/ga=26 and time=25	26	25	0.88	0.24	3.19
Dexa vs Placebo/ga=26 and time=26	26	26	1.00	0.24	4.16
Dexa vs Placebo/ga=26 and time=27	26	27	1.15	0.24	5.54
Dexa vs Placebo/ga=26 and time=28	26	28	1.33	0.24	7.56
Dexa vs Placebo/ga=27 and time=0	27	0	1.04	0.76	1.42
Dexa vs Placebo/ga=27 and time=1	27	1	0.91	0.68	1.21
Dexa vs Placebo/ga=27 and time=2	27	2	0.81	0.61	1.08
Dexa vs Placebo/ga=27 and time=3	27	3	0.72	0.53	0.99
Dexa vs Placebo/ga=27 and time=4	27	4	0.66	0.46	0.93
Dexa vs Placebo/ga=27 and time=5	27	5	0.60	0.40	0.89
Dexa vs Placebo/ga=27 and time=6	27	6	0.55	0.36	0.86
Dexa vs Placebo/ga=27 and time=7	27	7	0.52	0.32	0.84
Dexa vs Placebo/ga=27 and time=8	27	8	0.49	0.29	0.82
Dexa vs Placebo/ga=27 and time=9	27	9	0.47	0.27	0.80
Dexa vs Placebo/ga=27 and time=10	27	10	0.45	0.25	0.79

Dexa vs Placebo/ga=27 and time=11	27	11	0.44	0.24	0.79
Dexa vs Placebo/ga=27 and time=12	27	12	0.43	0.24	0.79
Dexa vs Placebo/ga=27 and time=13	27	13	0.43	0.23	0.79
Dexa vs Placebo/ga=27 and time=14	27	14	0.43	0.23	0.81
Dexa vs Placebo/ga=27 and time=15	27	15	0.44	0.23	0.83
Dexa vs Placebo/ga=27 and time=16	27	16	0.45	0.23	0.86
Dexa vs Placebo/ga=27 and time=17	27	17	0.46	0.23	0.91
Dexa vs Placebo/ga=27 and time=18	27	18	0.48	0.24	0.98
Dexa vs Placebo/ga=27 and time=19	27	19	0.51	0.24	1.08
Dexa vs Placebo/ga=27 and time=20	27	20	0.55	0.25	1.22
Dexa vs Placebo/ga=27 and time=21	27	21	0.59	0.25	1.40
Dexa vs Placebo/ga=27 and time=22	27	22	0.65	0.25	1.66
Dexa vs Placebo/ga=27 and time=23	27	23	0.71	0.25	2.01
Dexa vs Placebo/ga=27 and time=24	27	24	0.79	0.25	2.49
Dexa vs Placebo/ga=27 and time=25	27	25	0.89	0.25	3.17
Dexa vs Placebo/ga=27 and time=26	27	26	1.01	0.25	4.14
Dexa vs Placebo/ga=27 and time=27	27	27	1.16	0.25	5.52
Dexa vs Placebo/ga=27 and time=28	27	28	1.35	0.24	7.55
Dexa vs Placebo/ga=28 and time=0	28	0	1.05	0.80	1.37
Dexa vs Placebo/ga=28 and time=1	28	1	0.92	0.73	1.16
Dexa vs Placebo/ga=28 and time=2	28	2	0.82	0.65	1.03
Dexa vs Placebo/ga=28 and time=3	28	3	0.73	0.56	0.96
Dexa vs Placebo/ga=28 and time=4	28	4	0.66	0.48	0.91
Dexa vs Placebo/ga=28 and time=5	28	5	0.61	0.42	0.87
Dexa vs Placebo/ga=28 and time=6	28	6	0.56	0.37	0.85
Dexa vs Placebo/ga=28 and time=7	28	7	0.52	0.33	0.83
Dexa vs Placebo/ga=28 and time=8	28	8	0.50	0.30	0.81
Dexa vs Placebo/ga=28 and time=9	28	9	0.47	0.28	0.79
Dexa vs Placebo/ga=28 and time=10	28	10	0.45	0.26	0.79
Dexa vs Placebo/ga=28 and time=11	28	11	0.44	0.25	0.78
Dexa vs Placebo/ga=28 and time=12	28	12	0.44	0.24	0.78
Dexa vs Placebo/ga=28 and time=13	28	13	0.43	0.24	0.79
Dexa vs Placebo/ga=28 and time=14	28	14	0.43	0.24	0.80
Dexa vs Placebo/ga=28 and time=15	28	15	0.44	0.24	0.82
Dexa vs Placebo/ga=28 and time=16	28	16	0.45	0.24	0.86
Dexa vs Placebo/ga=28 and time=17	28	17	0.47	0.24	0.91
Dexa vs Placebo/ga=28 and time=18	28	18	0.49	0.25	0.98
Dexa vs Placebo/ga=28 and time=19	28	19	0.52	0.25	1.07
Dexa vs Placebo/ga=28 and time=20	28	20	0.55	0.25	1.21
Dexa vs Placebo/ga=28 and time=21	28	21	0.60	0.26	1.39
Dexa vs Placebo/ga=28 and time=22	28	22	0.65	0.26	1.65
Dexa vs Placebo/ga=28 and time=23	28	23	0.72	0.26	2.00
Dexa vs Placebo/ga=28 and time=24	28	24	0.80	0.26	2.49
Dexa vs Placebo/ga=28 and time=25	28	25	0.90	0.26	3.17
Dexa vs Placebo/ga=28 and time=26	28	26	1.02	0.25	4.13
Dexa vs Placebo/ga=28 and time=27	28	27	1.18	0.25	5.52
Dexa vs Placebo/ga=28 and time=28	28	28	1.37	0.25	7.55
Dexa vs Placebo/ga=29 and time=0	29	0	1.06	0.84	1.34

Dexa vs Placebo/ga=29 and time=1	29	1	0.93	0.77	1.13
Dexa vs Placebo/ga=29 and time=2	29	2	0.83	0.68	1.01
Dexa vs Placebo/ga=29 and time=3	29	3	0.74	0.59	0.94
Dexa vs Placebo/ga=29 and time=4	29	4	0.67	0.50	0.90
Dexa vs Placebo/ga=29 and time=5	29	5	0.61	0.44	0.87
Dexa vs Placebo/ga=29 and time=6	29	6	0.57	0.38	0.84
Dexa vs Placebo/ga=29 and time=7	29	7	0.53	0.34	0.82
Dexa vs Placebo/ga=29 and time=8	29	8	0.50	0.31	0.81
Dexa vs Placebo/ga=29 and time=9	29	9	0.48	0.29	0.79
Dexa vs Placebo/ga=29 and time=10	29	10	0.46	0.27	0.78
Dexa vs Placebo/ga=29 and time=11	29	11	0.45	0.26	0.78
Dexa vs Placebo/ga=29 and time=12	29	12	0.44	0.25	0.78
Dexa vs Placebo/ga=29 and time=13	29	13	0.44	0.24	0.79
Dexa vs Placebo/ga=29 and time=14	29	14	0.44	0.24	0.80
Dexa vs Placebo/ga=29 and time=15	29	15	0.45	0.24	0.82
Dexa vs Placebo/ga=29 and time=16	29	16	0.46	0.25	0.85
Dexa vs Placebo/ga=29 and time=17	29	17	0.47	0.25	0.90
Dexa vs Placebo/ga=29 and time=18	29	18	0.50	0.25	0.98
Dexa vs Placebo/ga=29 and time=19	29	19	0.52	0.26	1.07
Dexa vs Placebo/ga=29 and time=20	29	20	0.56	0.26	1.21
Dexa vs Placebo/ga=29 and time=21	29	21	0.61	0.26	1.40
Dexa vs Placebo/ga=29 and time=22	29	22	0.66	0.26	1.65
Dexa vs Placebo/ga=29 and time=23	29	23	0.73	0.26	2.00
Dexa vs Placebo/ga=29 and time=24	29	24	0.81	0.26	2.49
Dexa vs Placebo/ga=29 and time=25	29	25	0.91	0.26	3.18
Dexa vs Placebo/ga=29 and time=26	29	26	1.04	0.26	4.15
Dexa vs Placebo/ga=29 and time=27	29	27	1.19	0.26	5.54
Dexa vs Placebo/ga=29 and time=28	29	28	1.38	0.25	7.58
Dexa vs Placebo/ga=30 and time=0	30	0	1.07	0.87	1.33
Dexa vs Placebo/ga=30 and time=1	30	1	0.94	0.80	1.12
Dexa vs Placebo/ga=30 and time=2	30	2	0.84	0.70	1.00
Dexa vs Placebo/ga=30 and time=3	30	3	0.75	0.60	0.94
Dexa vs Placebo/ga=30 and time=4	30	4	0.68	0.51	0.90
Dexa vs Placebo/ga=30 and time=5	30	5	0.62	0.44	0.87
Dexa vs Placebo/ga=30 and time=6	30	6	0.58	0.39	0.85
Dexa vs Placebo/ga=30 and time=7	30	7	0.54	0.35	0.83
Dexa vs Placebo/ga=30 and time=8	30	8	0.51	0.32	0.81
Dexa vs Placebo/ga=30 and time=9	30	9	0.48	0.29	0.80
Dexa vs Placebo/ga=30 and time=10	30	10	0.47	0.27	0.79
Dexa vs Placebo/ga=30 and time=11	30	11	0.45	0.26	0.79
Dexa vs Placebo/ga=30 and time=12	30	12	0.45	0.25	0.79
Dexa vs Placebo/ga=30 and time=13	30	13	0.44	0.25	0.79
Dexa vs Placebo/ga=30 and time=14	30	14	0.45	0.25	0.81
Dexa vs Placebo/ga=30 and time=15	30	15	0.45	0.25	0.83
Dexa vs Placebo/ga=30 and time=16	30	16	0.46	0.25	0.86
Dexa vs Placebo/ga=30 and time=17	30	17	0.48	0.25	0.91
Dexa vs Placebo/ga=30 and time=18	30	18	0.50	0.26	0.98
Dexa vs Placebo/ga=30 and time=19	30	19	0.53	0.26	1.08

Dexa vs Placebo/ga=30 and time=20	30	20	0.57	0.26	1.22
Dexa vs Placebo/ga=30 and time=21	30	21	0.61	0.27	1.41
Dexa vs Placebo/ga=30 and time=22	30	22	0.67	0.27	1.66
Dexa vs Placebo/ga=30 and time=23	30	23	0.74	0.27	2.02
Dexa vs Placebo/ga=30 and time=24	30	24	0.82	0.27	2.51
Dexa vs Placebo/ga=30 and time=25	30	25	0.92	0.27	3.20
Dexa vs Placebo/ga=30 and time=26	30	26	1.05	0.26	4.17
Dexa vs Placebo/ga=30 and time=27	30	27	1.21	0.26	5.58
Dexa vs Placebo/ga=30 and time=28	30	28	1.40	0.26	7.64
Dexa vs Placebo/ga=31 and time=0	31	0	1.09	0.87	1.36
Dexa vs Placebo/ga=31 and time=1	31	1	0.96	0.80	1.15
Dexa vs Placebo/ga=31 and time=2	31	2	0.85	0.70	1.03
Dexa vs Placebo/ga=31 and time=3	31	3	0.76	0.60	0.96
Dexa vs Placebo/ga=31 and time=4	31	4	0.69	0.51	0.92
Dexa vs Placebo/ga=31 and time=5	31	5	0.63	0.45	0.89
Dexa vs Placebo/ga=31 and time=6	31	6	0.58	0.39	0.86
Dexa vs Placebo/ga=31 and time=7	31	7	0.54	0.35	0.84
Dexa vs Placebo/ga=31 and time=8	31	8	0.51	0.32	0.83
Dexa vs Placebo/ga=31 and time=9	31	9	0.49	0.29	0.82
Dexa vs Placebo/ga=31 and time=10	31	10	0.47	0.28	0.81
Dexa vs Placebo/ga=31 and time=11	31	11	0.46	0.26	0.80
Dexa vs Placebo/ga=31 and time=12	31	12	0.45	0.25	0.80
Dexa vs Placebo/ga=31 and time=13	31	13	0.45	0.25	0.81
Dexa vs Placebo/ga=31 and time=14	31	14	0.45	0.25	0.82
Dexa vs Placebo/ga=31 and time=15	31	15	0.46	0.25	0.84
Dexa vs Placebo/ga=31 and time=16	31	16	0.47	0.25	0.88
Dexa vs Placebo/ga=31 and time=17	31	17	0.49	0.25	0.93
Dexa vs Placebo/ga=31 and time=18	31	18	0.51	0.26	1.00
Dexa vs Placebo/ga=31 and time=19	31	19	0.54	0.26	1.10
Dexa vs Placebo/ga=31 and time=20	31	20	0.57	0.27	1.24
Dexa vs Placebo/ga=31 and time=21	31	21	0.62	0.27	1.42
Dexa vs Placebo/ga=31 and time=22	31	22	0.68	0.27	1.68
Dexa vs Placebo/ga=31 and time=23	31	23	0.75	0.27	2.04
Dexa vs Placebo/ga=31 and time=24	31	24	0.83	0.27	2.54
Dexa vs Placebo/ga=31 and time=25	31	25	0.93	0.27	3.23
Dexa vs Placebo/ga=31 and time=26	31	26	1.06	0.27	4.22
Dexa vs Placebo/ga=31 and time=27	31	27	1.22	0.26	5.64
Dexa vs Placebo/ga=31 and time=28	31	28	1.42	0.26	7.72
Dexa vs Placebo/ga=32 and time=0	32	0	1.10	0.85	1.42
Dexa vs Placebo/ga=32 and time=1	32	1	0.97	0.77	1.21
Dexa vs Placebo/ga=32 and time=2	32	2	0.86	0.68	1.08
Dexa vs Placebo/ga=32 and time=3	32	3	0.77	0.59	1.01
Dexa vs Placebo/ga=32 and time=4	32	4	0.70	0.51	0.96
Dexa vs Placebo/ga=32 and time=5	32	5	0.64	0.44	0.92
Dexa vs Placebo/ga=32 and time=6	32	6	0.59	0.39	0.89
Dexa vs Placebo/ga=32 and time=7	32	7	0.55	0.35	0.87
Dexa vs Placebo/ga=32 and time=8	32	8	0.52	0.32	0.85
Dexa vs Placebo/ga=32 and time=9	32	9	0.50	0.29	0.84

Dexa vs Placebo/ga=32 and time=10	32	10	0.48	0.27	0.83
Dexa vs Placebo/ga=32 and time=11	32	11	0.46	0.26	0.82
Dexa vs Placebo/ga=32 and time=12	32	12	0.46	0.25	0.82
Dexa vs Placebo/ga=32 and time=13	32	13	0.45	0.25	0.83
Dexa vs Placebo/ga=32 and time=14	32	14	0.46	0.25	0.84
Dexa vs Placebo/ga=32 and time=15	32	15	0.46	0.25	0.86
Dexa vs Placebo/ga=32 and time=16	32	16	0.47	0.25	0.90
Dexa vs Placebo/ga=32 and time=17	32	17	0.49	0.25	0.95
Dexa vs Placebo/ga=32 and time=18	32	18	0.51	0.26	1.02
Dexa vs Placebo/ga=32 and time=19	32	19	0.54	0.26	1.12
Dexa vs Placebo/ga=32 and time=20	32	20	0.58	0.27	1.26
Dexa vs Placebo/ga=32 and time=21	32	21	0.63	0.27	1.45
Dexa vs Placebo/ga=32 and time=22	32	22	0.68	0.27	1.72
Dexa vs Placebo/ga=32 and time=23	32	23	0.75	0.27	2.08
Dexa vs Placebo/ga=32 and time=24	32	24	0.84	0.27	2.58
Dexa vs Placebo/ga=32 and time=25	32	25	0.95	0.27	3.29
Dexa vs Placebo/ga=32 and time=26	32	26	1.08	0.27	4.28
Dexa vs Placebo/ga=32 and time=27	32	27	1.23	0.27	5.72
Dexa vs Placebo/ga=32 and time=28	32	28	1.43	0.26	7.82
Dexa vs Placebo/ga=33 and time=0	33	0	1.11	0.82	1.51
Dexa vs Placebo/ga=33 and time=1	33	1	0.98	0.74	1.29
Dexa vs Placebo/ga=33 and time=2	33	2	0.87	0.65	1.15
Dexa vs Placebo/ga=33 and time=3	33	3	0.78	0.57	1.07
Dexa vs Placebo/ga=33 and time=4	33	4	0.70	0.49	1.01
Dexa vs Placebo/ga=33 and time=5	33	5	0.64	0.43	0.97
Dexa vs Placebo/ga=33 and time=6	33	6	0.60	0.38	0.94
Dexa vs Placebo/ga=33 and time=7	33	7	0.56	0.34	0.91
Dexa vs Placebo/ga=33 and time=8	33	8	0.53	0.31	0.89
Dexa vs Placebo/ga=33 and time=9	33	9	0.50	0.29	0.87
Dexa vs Placebo/ga=33 and time=10	33	10	0.48	0.27	0.86
Dexa vs Placebo/ga=33 and time=11	33	11	0.47	0.26	0.86
Dexa vs Placebo/ga=33 and time=12	33	12	0.46	0.25	0.85
Dexa vs Placebo/ga=33 and time=13	33	13	0.46	0.25	0.86
Dexa vs Placebo/ga=33 and time=14	33	14	0.46	0.24	0.87
Dexa vs Placebo/ga=33 and time=15	33	15	0.47	0.25	0.89
Dexa vs Placebo/ga=33 and time=16	33	16	0.48	0.25	0.93
Dexa vs Placebo/ga=33 and time=17	33	17	0.50	0.25	0.98
Dexa vs Placebo/ga=33 and time=18	33	18	0.52	0.26	1.05
Dexa vs Placebo/ga=33 and time=19	33	19	0.55	0.26	1.16
Dexa vs Placebo/ga=33 and time=20	33	20	0.59	0.27	1.30
Dexa vs Placebo/ga=33 and time=21	33	21	0.64	0.27	1.49
Dexa vs Placebo/ga=33 and time=22	33	22	0.69	0.27	1.76
Dexa vs Placebo/ga=33 and time=23	33	23	0.76	0.27	2.13
Dexa vs Placebo/ga=33 and time=24	33	24	0.85	0.27	2.64
Dexa vs Placebo/ga=33 and time=25	33	25	0.96	0.27	3.35
Dexa vs Placebo/ga=33 and time=26	33	26	1.09	0.27	4.37
Dexa vs Placebo/ga=33 and time=27	33	27	1.25	0.27	5.82
Dexa vs Placebo/ga=33 and time=28	33	28	1.45	0.26	7.95

Supplementary Table 1b. Neonatal death (0 - 24 hours)

Label	Gestational age at first dose, weeks	Administration-to-birth interval, hours	RR	Lower 95%CL	Upper 95%CL
Dexa vs Placebo/ga=26 and time in hours=0	26	0	1.02	0.70	1.49
Dexa vs Placebo/ga=26 and time in hours=1	26	1	1.02	0.70	1.48
Dexa vs Placebo/ga=26 and time in hours=2	26	2	1.01	0.70	1.47
Dexa vs Placebo/ga=26 and time in hours=3	26	3	1.01	0.70	1.46
Dexa vs Placebo/ga=26 and time in hours=4	26	4	1.00	0.69	1.45
Dexa vs Placebo/ga=26 and time in hours=5	26	5	1.00	0.69	1.44
Dexa vs Placebo/ga=26 and time in hours=6	26	6	0.99	0.69	1.43
Dexa vs Placebo/ga=26 and time in hours=7	26	7	0.99	0.68	1.42
Dexa vs Placebo/ga=26 and time in hours=8	26	8	0.98	0.68	1.41
Dexa vs Placebo/ga=26 and time in hours=9	26	9	0.97	0.68	1.40
Dexa vs Placebo/ga=26 and time in hours=10	26	10	0.97	0.68	1.39
Dexa vs Placebo/ga=26 and time in hours=11	26	11	0.96	0.67	1.38
Dexa vs Placebo/ga=26 and time in hours=12	26	12	0.96	0.67	1.37
Dexa vs Placebo/ga=26 and time in hours=13	26	13	0.95	0.67	1.36
Dexa vs Placebo/ga=26 and time in hours=14	26	14	0.95	0.66	1.35
Dexa vs Placebo/ga=26 and time in hours=15	26	15	0.94	0.66	1.35
Dexa vs Placebo/ga=26 and time in hours=16	26	16	0.94	0.66	1.34
Dexa vs Placebo/ga=26 and time in hours=17	26	17	0.93	0.66	1.33
Dexa vs Placebo/ga=26 and time in hours=18	26	18	0.93	0.65	1.32
Dexa vs Placebo/ga=26 and time in hours=19	26	19	0.92	0.65	1.31
Dexa vs Placebo/ga=26 and time in hours=20	26	20	0.92	0.65	1.30
Dexa vs Placebo/ga=26 and time in hours=21	26	21	0.91	0.64	1.30
Dexa vs Placebo/ga=26 and time in hours=22	26	22	0.91	0.64	1.29
Dexa vs Placebo/ga=26 and time in hours=23	26	23	0.90	0.64	1.28
Dexa vs Placebo/ga=26 and time in hours=24	26	24	0.90	0.64	1.27
Dexa vs Placebo/ga=27 and time in hours=0	27	0	1.04	0.76	1.42
Dexa vs Placebo/ga=27 and time in hours=1	27	1	1.03	0.75	1.41
Dexa vs Placebo/ga=27 and time in hours=2	27	2	1.03	0.75	1.40
Dexa vs Placebo/ga=27 and time in hours=3	27	3	1.02	0.75	1.39
Dexa vs Placebo/ga=27 and time in hours=4	27	4	1.01	0.74	1.38
Dexa vs Placebo/ga=27 and time in hours=5	27	5	1.01	0.74	1.37
Dexa vs Placebo/ga=27 and time in hours=6	27	6	1.00	0.74	1.36
Dexa vs Placebo/ga=27 and time in hours=7	27	7	1.00	0.74	1.35
Dexa vs Placebo/ga=27 and time in hours=8	27	8	0.99	0.73	1.34
Dexa vs Placebo/ga=27 and time in hours=9	27	9	0.99	0.73	1.33
Dexa vs Placebo/ga=27 and time in hours=10	27	10	0.98	0.73	1.32
Dexa vs Placebo/ga=27 and time in hours=11	27	11	0.98	0.72	1.31
Dexa vs Placebo/ga=27 and time in hours=12	27	12	0.97	0.72	1.31
Dexa vs Placebo/ga=27 and time in hours=13	27	13	0.97	0.72	1.30
Dexa vs Placebo/ga=27 and time in hours=14	27	14	0.96	0.72	1.29
Dexa vs Placebo/ga=27 and time in hours=15	27	15	0.96	0.71	1.28
Dexa vs Placebo/ga=27 and time in hours=16	27	16	0.95	0.71	1.27
Dexa vs Placebo/ga=27 and time in hours=17	27	17	0.94	0.71	1.26
Dexa vs Placebo/ga=27 and time in hours=18	27	18	0.94	0.70	1.26
Dexa vs Placebo/ga=27 and time in hours=19	27	19	0.93	0.70	1.25
Dexa vs Placebo/ga=27 and time in hours=20	27	20	0.93	0.70	1.24

Dexa vs Placebo/ga=27 and time in hours=21	27	21	0.93	0.69	1.23
Dexa vs Placebo/ga=27 and time in hours=22	27	22	0.92	0.69	1.23
Dexa vs Placebo/ga=27 and time in hours=23	27	23	0.92	0.69	1.22
Dexa vs Placebo/ga=27 and time in hours=24	27	24	0.91	0.68	1.21
Dexa vs Placebo/ga=28 and time in hours=0	28	0	1.05	0.80	1.37
Dexa vs Placebo/ga=28 and time in hours=1	28	1	1.04	0.80	1.36
Dexa vs Placebo/ga=28 and time in hours=2	28	2	1.04	0.80	1.35
Dexa vs Placebo/ga=28 and time in hours=3	28	3	1.03	0.80	1.34
Dexa vs Placebo/ga=28 and time in hours=4	28	4	1.03	0.79	1.33
Dexa vs Placebo/ga=28 and time in hours=5	28	5	1.02	0.79	1.32
Dexa vs Placebo/ga=28 and time in hours=6	28	6	1.02	0.79	1.31
Dexa vs Placebo/ga=28 and time in hours=7	28	7	1.01	0.78	1.30
Dexa vs Placebo/ga=28 and time in hours=8	28	8	1.00	0.78	1.29
Dexa vs Placebo/ga=28 and time in hours=9	28	9	1.00	0.78	1.28
Dexa vs Placebo/ga=28 and time in hours=10	28	10	0.99	0.78	1.27
Dexa vs Placebo/ga=28 and time in hours=11	28	11	0.99	0.77	1.26
Dexa vs Placebo/ga=28 and time in hours=12	28	12	0.98	0.77	1.25
Dexa vs Placebo/ga=28 and time in hours=13	28	13	0.98	0.77	1.24
Dexa vs Placebo/ga=28 and time in hours=14	28	14	0.97	0.76	1.24
Dexa vs Placebo/ga=28 and time in hours=15	28	15	0.97	0.76	1.23
Dexa vs Placebo/ga=28 and time in hours=16	28	16	0.96	0.76	1.22
Dexa vs Placebo/ga=28 and time in hours=17	28	17	0.96	0.75	1.21
Dexa vs Placebo/ga=28 and time in hours=18	28	18	0.95	0.75	1.20
Dexa vs Placebo/ga=28 and time in hours=19	28	19	0.95	0.75	1.20
Dexa vs Placebo/ga=28 and time in hours=20	28	20	0.94	0.75	1.19
Dexa vs Placebo/ga=28 and time in hours=21	28	21	0.94	0.74	1.18
Dexa vs Placebo/ga=28 and time in hours=22	28	22	0.93	0.74	1.17
Dexa vs Placebo/ga=28 and time in hours=23	28	23	0.93	0.74	1.17
Dexa vs Placebo/ga=28 and time in hours=24	28	24	0.92	0.73	1.16
Dexa vs Placebo/ga=29 and time in hours=0	29	0	1.06	0.84	1.34
Dexa vs Placebo/ga=29 and time in hours=1	29	1	1.06	0.84	1.33
Dexa vs Placebo/ga=29 and time in hours=2	29	2	1.05	0.84	1.32
Dexa vs Placebo/ga=29 and time in hours=3	29	3	1.04	0.84	1.31
Dexa vs Placebo/ga=29 and time in hours=4	29	4	1.04	0.83	1.29
Dexa vs Placebo/ga=29 and time in hours=5	29	5	1.03	0.83	1.28
Dexa vs Placebo/ga=29 and time in hours=6	29	6	1.03	0.83	1.27
Dexa vs Placebo/ga=29 and time in hours=7	29	7	1.02	0.83	1.26
Dexa vs Placebo/ga=29 and time in hours=8	29	8	1.02	0.82	1.26
Dexa vs Placebo/ga=29 and time in hours=9	29	9	1.01	0.82	1.25
Dexa vs Placebo/ga=29 and time in hours=10	29	10	1.00	0.82	1.24
Dexa vs Placebo/ga=29 and time in hours=11	29	11	1.00	0.81	1.23
Dexa vs Placebo/ga=29 and time in hours=12	29	12	0.99	0.81	1.22
Dexa vs Placebo/ga=29 and time in hours=13	29	13	0.99	0.81	1.21
Dexa vs Placebo/ga=29 and time in hours=14	29	14	0.98	0.80	1.20
Dexa vs Placebo/ga=29 and time in hours=15	29	15	0.98	0.80	1.19
Dexa vs Placebo/ga=29 and time in hours=16	29	16	0.97	0.80	1.18
Dexa vs Placebo/ga=29 and time in hours=17	29	17	0.97	0.80	1.18
Dexa vs Placebo/ga=29 and time in hours=18	29	18	0.96	0.79	1.17
Dexa vs Placebo/ga=29 and time in hours=19	29	19	0.96	0.79	1.16
Dexa vs Placebo/ga=29 and time in hours=20	29	20	0.95	0.79	1.15
Dexa vs Placebo/ga=29 and time in hours=21	29	21	0.95	0.78	1.15
Dexa vs Placebo/ga=29 and time in hours=22	29	22	0.94	0.78	1.14

Dexa vs Placebo/ga=29 and time in hours=23	29	23	0.94	0.78	1.13
Dexa vs Placebo/ga=29 and time in hours=24	29	24	0.93	0.77	1.13
Dexa vs Placebo/ga=30 and time in hours=0	30	0	1.07	0.87	1.33
Dexa vs Placebo/ga=30 and time in hours=1	30	1	1.07	0.86	1.32
Dexa vs Placebo/ga=30 and time in hours=2	30	2	1.06	0.86	1.31
Dexa vs Placebo/ga=30 and time in hours=3	30	3	1.06	0.86	1.30
Dexa vs Placebo/ga=30 and time in hours=4	30	4	1.05	0.86	1.29
Dexa vs Placebo/ga=30 and time in hours=5	30	5	1.05	0.85	1.28
Dexa vs Placebo/ga=30 and time in hours=6	30	6	1.04	0.85	1.27
Dexa vs Placebo/ga=30 and time in hours=7	30	7	1.03	0.85	1.26
Dexa vs Placebo/ga=30 and time in hours=8	30	8	1.03	0.85	1.25
Dexa vs Placebo/ga=30 and time in hours=9	30	9	1.02	0.84	1.24
Dexa vs Placebo/ga=30 and time in hours=10	30	10	1.02	0.84	1.23
Dexa vs Placebo/ga=30 and time in hours=11	30	11	1.01	0.84	1.22
Dexa vs Placebo/ga=30 and time in hours=12	30	12	1.01	0.84	1.21
Dexa vs Placebo/ga=30 and time in hours=13	30	13	1.00	0.83	1.20
Dexa vs Placebo/ga=30 and time in hours=14	30	14	1.00	0.83	1.19
Dexa vs Placebo/ga=30 and time in hours=15	30	15	0.99	0.83	1.19
Dexa vs Placebo/ga=30 and time in hours=16	30	16	0.98	0.82	1.18
Dexa vs Placebo/ga=30 and time in hours=17	30	17	0.98	0.82	1.17
Dexa vs Placebo/ga=30 and time in hours=18	30	18	0.97	0.82	1.16
Dexa vs Placebo/ga=30 and time in hours=19	30	19	0.97	0.81	1.15
Dexa vs Placebo/ga=30 and time in hours=20	30	20	0.96	0.81	1.15
Dexa vs Placebo/ga=30 and time in hours=21	30	21	0.96	0.81	1.14
Dexa vs Placebo/ga=30 and time in hours=22	30	22	0.95	0.80	1.13
Dexa vs Placebo/ga=30 and time in hours=23	30	23	0.95	0.80	1.13
Dexa vs Placebo/ga=30 and time in hours=24	30	24	0.94	0.80	1.12
Dexa vs Placebo/ga=31 and time in hours=0	31	0	1.09	0.87	1.36
Dexa vs Placebo/ga=31 and time in hours=1	31	1	1.08	0.87	1.35
Dexa vs Placebo/ga=31 and time in hours=2	31	2	1.08	0.86	1.34
Dexa vs Placebo/ga=31 and time in hours=3	31	3	1.07	0.86	1.33
Dexa vs Placebo/ga=31 and time in hours=4	31	4	1.06	0.86	1.32
Dexa vs Placebo/ga=31 and time in hours=5	31	5	1.06	0.86	1.31
Dexa vs Placebo/ga=31 and time in hours=6	31	6	1.05	0.85	1.30
Dexa vs Placebo/ga=31 and time in hours=7	31	7	1.05	0.85	1.29
Dexa vs Placebo/ga=31 and time in hours=8	31	8	1.04	0.85	1.28
Dexa vs Placebo/ga=31 and time in hours=9	31	9	1.03	0.84	1.27
Dexa vs Placebo/ga=31 and time in hours=10	31	10	1.03	0.84	1.26
Dexa vs Placebo/ga=31 and time in hours=11	31	11	1.02	0.84	1.25
Dexa vs Placebo/ga=31 and time in hours=12	31	12	1.02	0.84	1.24
Dexa vs Placebo/ga=31 and time in hours=13	31	13	1.01	0.83	1.23
Dexa vs Placebo/ga=31 and time in hours=14	31	14	1.01	0.83	1.22
Dexa vs Placebo/ga=31 and time in hours=15	31	15	1.00	0.83	1.21
Dexa vs Placebo/ga=31 and time in hours=16	31	16	1.00	0.82	1.21
Dexa vs Placebo/ga=31 and time in hours=17	31	17	0.99	0.82	1.20
Dexa vs Placebo/ga=31 and time in hours=18	31	18	0.99	0.82	1.19
Dexa vs Placebo/ga=31 and time in hours=19	31	19	0.98	0.81	1.18
Dexa vs Placebo/ga=31 and time in hours=20	31	20	0.98	0.81	1.18
Dexa vs Placebo/ga=31 and time in hours=21	31	21	0.97	0.81	1.17
Dexa vs Placebo/ga=31 and time in hours=22	31	22	0.97	0.80	1.16
Dexa vs Placebo/ga=31 and time in hours=23	31	23	0.96	0.80	1.15
Dexa vs Placebo/ga=31 and time in hours=24	31	24	0.96	0.80	1.15

Dexa vs Placebo/ga=32 and time in hours=0	32	0	1.10	0.85	1.42
Dexa vs Placebo/ga=32 and time in hours=1	32	1	1.09	0.85	1.41
Dexa vs Placebo/ga=32 and time in hours=2	32	2	1.09	0.85	1.40
Dexa vs Placebo/ga=32 and time in hours=3	32	3	1.08	0.84	1.39
Dexa vs Placebo/ga=32 and time in hours=4	32	4	1.08	0.84	1.38
Dexa vs Placebo/ga=32 and time in hours=5	32	5	1.07	0.84	1.37
Dexa vs Placebo/ga=32 and time in hours=6	32	6	1.06	0.84	1.36
Dexa vs Placebo/ga=32 and time in hours=7	32	7	1.06	0.83	1.35
Dexa vs Placebo/ga=32 and time in hours=8	32	8	1.05	0.83	1.34
Dexa vs Placebo/ga=32 and time in hours=9	32	9	1.05	0.83	1.33
Dexa vs Placebo/ga=32 and time in hours=10	32	10	1.04	0.82	1.32
Dexa vs Placebo/ga=32 and time in hours=11	32	11	1.04	0.82	1.31
Dexa vs Placebo/ga=32 and time in hours=12	32	12	1.03	0.82	1.30
Dexa vs Placebo/ga=32 and time in hours=13	32	13	1.02	0.81	1.29
Dexa vs Placebo/ga=32 and time in hours=14	32	14	1.02	0.81	1.28
Dexa vs Placebo/ga=32 and time in hours=15	32	15	1.01	0.81	1.27
Dexa vs Placebo/ga=32 and time in hours=16	32	16	1.01	0.80	1.27
Dexa vs Placebo/ga=32 and time in hours=17	32	17	1.00	0.80	1.26
Dexa vs Placebo/ga=32 and time in hours=18	32	18	1.00	0.80	1.25
Dexa vs Placebo/ga=32 and time in hours=19	32	19	0.99	0.79	1.24
Dexa vs Placebo/ga=32 and time in hours=20	32	20	0.99	0.79	1.24
Dexa vs Placebo/ga=32 and time in hours=21	32	21	0.98	0.79	1.23
Dexa vs Placebo/ga=32 and time in hours=22	32	22	0.98	0.78	1.22
Dexa vs Placebo/ga=32 and time in hours=23	32	23	0.97	0.78	1.21
Dexa vs Placebo/ga=32 and time in hours=24	32	24	0.97	0.77	1.21
Dexa vs Placebo/ga=33 and time in hours=0	33	0	1.11	0.82	1.51
Dexa vs Placebo/ga=33 and time in hours=1	33	1	1.11	0.82	1.49
Dexa vs Placebo/ga=33 and time in hours=2	33	2	1.10	0.82	1.48
Dexa vs Placebo/ga=33 and time in hours=3	33	3	1.10	0.81	1.47
Dexa vs Placebo/ga=33 and time in hours=4	33	4	1.09	0.81	1.46
Dexa vs Placebo/ga=33 and time in hours=5	33	5	1.08	0.81	1.45
Dexa vs Placebo/ga=33 and time in hours=6	33	6	1.08	0.81	1.44
Dexa vs Placebo/ga=33 and time in hours=7	33	7	1.07	0.80	1.43
Dexa vs Placebo/ga=33 and time in hours=8	33	8	1.07	0.80	1.42
Dexa vs Placebo/ga=33 and time in hours=9	33	9	1.06	0.80	1.41
Dexa vs Placebo/ga=33 and time in hours=10	33	10	1.05	0.79	1.40
Dexa vs Placebo/ga=33 and time in hours=11	33	11	1.05	0.79	1.39
Dexa vs Placebo/ga=33 and time in hours=12	33	12	1.04	0.78	1.38
Dexa vs Placebo/ga=33 and time in hours=13	33	13	1.04	0.78	1.38
Dexa vs Placebo/ga=33 and time in hours=14	33	14	1.03	0.78	1.37
Dexa vs Placebo/ga=33 and time in hours=15	33	15	1.03	0.77	1.36
Dexa vs Placebo/ga=33 and time in hours=16	33	16	1.02	0.77	1.35
Dexa vs Placebo/ga=33 and time in hours=17	33	17	1.01	0.77	1.34
Dexa vs Placebo/ga=33 and time in hours=18	33	18	1.01	0.76	1.33
Dexa vs Placebo/ga=33 and time in hours=19	33	19	1.00	0.76	1.33
Dexa vs Placebo/ga=33 and time in hours=20	33	20	1.00	0.76	1.32
Dexa vs Placebo/ga=33 and time in hours=21	33	21	0.99	0.75	1.31
Dexa vs Placebo/ga=33 and time in hours=22	33	22	0.99	0.75	1.30
Dexa vs Placebo/ga=33 and time in hours=23	33	23	0.98	0.75	1.30
Dexa vs Placebo/ga=33 and time in hours=24	33	24	0.98	0.74	1.29

Supplementary Table 1c. Any baby death(0-28d)

Label	Gestational age at first dose, weeks	Administration-to-birth interval, days	RR	Lower 95%CL	Upper 95%CL
Dexa vs Placebo/ga=26 and time=0	26	0	0.99	0.72	1.38
Dexa vs Placebo/ga=26 and time=1	26	1	0.93	0.69	1.25
Dexa vs Placebo/ga=26 and time=2	26	2	0.87	0.65	1.17
Dexa vs Placebo/ga=26 and time=3	26	3	0.82	0.61	1.11
Dexa vs Placebo/ga=26 and time=4	26	4	0.78	0.56	1.08
Dexa vs Placebo/ga=26 and time=5	26	5	0.75	0.53	1.05
Dexa vs Placebo/ga=26 and time=6	26	6	0.72	0.49	1.04
Dexa vs Placebo/ga=26 and time=7	26	7	0.69	0.47	1.03
Dexa vs Placebo/ga=26 and time=8	26	8	0.68	0.45	1.03
Dexa vs Placebo/ga=26 and time=9	26	9	0.67	0.43	1.03
Dexa vs Placebo/ga=26 and time=10	26	10	0.66	0.42	1.03
Dexa vs Placebo/ga=26 and time=11	26	11	0.65	0.41	1.04
Dexa vs Placebo/ga=26 and time=12	26	12	0.65	0.41	1.05
Dexa vs Placebo/ga=26 and time=13	26	13	0.66	0.41	1.07
Dexa vs Placebo/ga=26 and time=14	26	14	0.67	0.41	1.09
Dexa vs Placebo/ga=26 and time=15	26	15	0.68	0.41	1.13
Dexa vs Placebo/ga=26 and time=16	26	16	0.70	0.42	1.17
Dexa vs Placebo/ga=26 and time=17	26	17	0.72	0.42	1.23
Dexa vs Placebo/ga=26 and time=18	26	18	0.75	0.43	1.32
Dexa vs Placebo/ga=26 and time=19	26	19	0.79	0.44	1.43
Dexa vs Placebo/ga=26 and time=20	26	20	0.83	0.44	1.57
Dexa vs Placebo/ga=26 and time=21	26	21	0.88	0.44	1.76
Dexa vs Placebo/ga=26 and time=22	26	22	0.94	0.44	2.00
Dexa vs Placebo/ga=26 and time=23	26	23	1.01	0.44	2.32
Dexa vs Placebo/ga=26 and time=24	26	24	1.09	0.44	2.75
Dexa vs Placebo/ga=26 and time=25	26	25	1.19	0.43	3.30
Dexa vs Placebo/ga=26 and time=26	26	26	1.30	0.42	4.04
Dexa vs Placebo/ga=26 and time=27	26	27	1.44	0.41	5.02
Dexa vs Placebo/ga=26 and time=28	26	28	1.60	0.40	6.35
Dexa vs Placebo/ga=27 and time=0	27	0	1.01	0.76	1.33
Dexa vs Placebo/ga=27 and time=1	27	1	0.94	0.73	1.20
Dexa vs Placebo/ga=27 and time=2	27	2	0.88	0.69	1.12
Dexa vs Placebo/ga=27 and time=3	27	3	0.83	0.65	1.07
Dexa vs Placebo/ga=27 and time=4	27	4	0.79	0.60	1.04
Dexa vs Placebo/ga=27 and time=5	27	5	0.76	0.56	1.03
Dexa vs Placebo/ga=27 and time=6	27	6	0.73	0.52	1.02
Dexa vs Placebo/ga=27 and time=7	27	7	0.70	0.49	1.01
Dexa vs Placebo/ga=27 and time=8	27	8	0.69	0.47	1.01
Dexa vs Placebo/ga=27 and time=9	27	9	0.67	0.45	1.01
Dexa vs Placebo/ga=27 and time=10	27	10	0.67	0.44	1.02
Dexa vs Placebo/ga=27 and time=11	27	11	0.66	0.43	1.03
Dexa vs Placebo/ga=27 and time=12	27	12	0.66	0.42	1.04
Dexa vs Placebo/ga=27 and time=13	27	13	0.67	0.42	1.06
Dexa vs Placebo/ga=27 and time=14	27	14	0.68	0.42	1.08

Dexa vs Placebo/ga=27 and time=15	27	15	0.69	0.43	1.11
Dexa vs Placebo/ga=27 and time=16	27	16	0.71	0.43	1.16
Dexa vs Placebo/ga=27 and time=17	27	17	0.73	0.44	1.22
Dexa vs Placebo/ga=27 and time=18	27	18	0.76	0.45	1.30
Dexa vs Placebo/ga=27 and time=19	27	19	0.80	0.45	1.41
Dexa vs Placebo/ga=27 and time=20	27	20	0.84	0.46	1.55
Dexa vs Placebo/ga=27 and time=21	27	21	0.89	0.46	1.74
Dexa vs Placebo/ga=27 and time=22	27	22	0.95	0.46	1.99
Dexa vs Placebo/ga=27 and time=23	27	23	1.02	0.45	2.31
Dexa vs Placebo/ga=27 and time=24	27	24	1.11	0.45	2.74
Dexa vs Placebo/ga=27 and time=25	27	25	1.21	0.44	3.29
Dexa vs Placebo/ga=27 and time=26	27	26	1.32	0.43	4.03
Dexa vs Placebo/ga=27 and time=27	27	27	1.46	0.42	5.02
Dexa vs Placebo/ga=27 and time=28	27	28	1.62	0.41	6.35
Dexa vs Placebo/ga=28 and time=0	28	0	1.02	0.81	1.29
Dexa vs Placebo/ga=28 and time=1	28	1	0.95	0.78	1.16
Dexa vs Placebo/ga=28 and time=2	28	2	0.89	0.73	1.09
Dexa vs Placebo/ga=28 and time=3	28	3	0.84	0.68	1.04
Dexa vs Placebo/ga=28 and time=4	28	4	0.80	0.63	1.02
Dexa vs Placebo/ga=28 and time=5	28	5	0.77	0.58	1.01
Dexa vs Placebo/ga=28 and time=6	28	6	0.74	0.54	1.00
Dexa vs Placebo/ga=28 and time=7	28	7	0.71	0.51	1.00
Dexa vs Placebo/ga=28 and time=8	28	8	0.70	0.48	1.00
Dexa vs Placebo/ga=28 and time=9	28	9	0.68	0.47	1.00
Dexa vs Placebo/ga=28 and time=10	28	10	0.68	0.45	1.01
Dexa vs Placebo/ga=28 and time=11	28	11	0.67	0.44	1.02
Dexa vs Placebo/ga=28 and time=12	28	12	0.67	0.44	1.03
Dexa vs Placebo/ga=28 and time=13	28	13	0.68	0.44	1.05
Dexa vs Placebo/ga=28 and time=14	28	14	0.69	0.44	1.07
Dexa vs Placebo/ga=28 and time=15	28	15	0.70	0.44	1.11
Dexa vs Placebo/ga=28 and time=16	28	16	0.72	0.45	1.15
Dexa vs Placebo/ga=28 and time=17	28	17	0.74	0.46	1.21
Dexa vs Placebo/ga=28 and time=18	28	18	0.77	0.46	1.30
Dexa vs Placebo/ga=28 and time=19	28	19	0.81	0.47	1.40
Dexa vs Placebo/ga=28 and time=20	28	20	0.85	0.47	1.55
Dexa vs Placebo/ga=28 and time=21	28	21	0.91	0.47	1.74
Dexa vs Placebo/ga=28 and time=22	28	22	0.97	0.47	1.99
Dexa vs Placebo/ga=28 and time=23	28	23	1.04	0.47	2.31
Dexa vs Placebo/ga=28 and time=24	28	24	1.12	0.46	2.74
Dexa vs Placebo/ga=28 and time=25	28	25	1.22	0.45	3.30
Dexa vs Placebo/ga=28 and time=26	28	26	1.34	0.44	4.04
Dexa vs Placebo/ga=28 and time=27	28	27	1.48	0.43	5.03
Dexa vs Placebo/ga=28 and time=28	28	28	1.64	0.42	6.37
Dexa vs Placebo/ga=29 and time=0	29	0	1.04	0.84	1.27
Dexa vs Placebo/ga=29 and time=1	29	1	0.97	0.82	1.14
Dexa vs Placebo/ga=29 and time=2	29	2	0.91	0.77	1.06
Dexa vs Placebo/ga=29 and time=3	29	3	0.85	0.71	1.03
Dexa vs Placebo/ga=29 and time=4	29	4	0.81	0.65	1.01
Dexa vs Placebo/ga=29 and time=5	29	5	0.78	0.60	1.00
Dexa vs Placebo/ga=29 and time=6	29	6	0.75	0.56	1.00
Dexa vs Placebo/ga=29 and time=7	29	7	0.72	0.53	1.00
Dexa vs Placebo/ga=29 and time=8	29	8	0.71	0.50	1.00

Dexa vs Placebo/ga=29 and time=9	29	9	0.69	0.48	1.00
Dexa vs Placebo/ga=29 and time=10	29	10	0.68	0.46	1.01
Dexa vs Placebo/ga=29 and time=11	29	11	0.68	0.45	1.02
Dexa vs Placebo/ga=29 and time=12	29	12	0.68	0.45	1.03
Dexa vs Placebo/ga=29 and time=13	29	13	0.69	0.45	1.05
Dexa vs Placebo/ga=29 and time=14	29	14	0.70	0.45	1.08
Dexa vs Placebo/ga=29 and time=15	29	15	0.71	0.45	1.11
Dexa vs Placebo/ga=29 and time=16	29	16	0.73	0.46	1.15
Dexa vs Placebo/ga=29 and time=17	29	17	0.75	0.47	1.22
Dexa vs Placebo/ga=29 and time=18	29	18	0.78	0.47	1.30
Dexa vs Placebo/ga=29 and time=19	29	19	0.82	0.48	1.41
Dexa vs Placebo/ga=29 and time=20	29	20	0.87	0.48	1.55
Dexa vs Placebo/ga=29 and time=21	29	21	0.92	0.48	1.75
Dexa vs Placebo/ga=29 and time=22	29	22	0.98	0.48	2.00
Dexa vs Placebo/ga=29 and time=23	29	23	1.05	0.48	2.32
Dexa vs Placebo/ga=29 and time=24	29	24	1.14	0.47	2.75
Dexa vs Placebo/ga=29 and time=25	29	25	1.24	0.46	3.31
Dexa vs Placebo/ga=29 and time=26	29	26	1.36	0.45	4.06
Dexa vs Placebo/ga=29 and time=27	29	27	1.50	0.44	5.06
Dexa vs Placebo/ga=29 and time=28	29	28	1.66	0.43	6.42
Dexa vs Placebo/ga=30 and time=0	30	0	1.05	0.87	1.27
Dexa vs Placebo/ga=30 and time=1	30	1	0.98	0.84	1.14
Dexa vs Placebo/ga=30 and time=2	30	2	0.92	0.79	1.06
Dexa vs Placebo/ga=30 and time=3	30	3	0.87	0.73	1.03
Dexa vs Placebo/ga=30 and time=4	30	4	0.82	0.67	1.01
Dexa vs Placebo/ga=30 and time=5	30	5	0.79	0.61	1.01
Dexa vs Placebo/ga=30 and time=6	30	6	0.76	0.57	1.01
Dexa vs Placebo/ga=30 and time=7	30	7	0.73	0.53	1.01
Dexa vs Placebo/ga=30 and time=8	30	8	0.72	0.51	1.01
Dexa vs Placebo/ga=30 and time=9	30	9	0.70	0.49	1.02
Dexa vs Placebo/ga=30 and time=10	30	10	0.69	0.47	1.02
Dexa vs Placebo/ga=30 and time=11	30	11	0.69	0.46	1.03
Dexa vs Placebo/ga=30 and time=12	30	12	0.69	0.46	1.05
Dexa vs Placebo/ga=30 and time=13	30	13	0.70	0.46	1.06
Dexa vs Placebo/ga=30 and time=14	30	14	0.71	0.46	1.09
Dexa vs Placebo/ga=30 and time=15	30	15	0.72	0.46	1.12
Dexa vs Placebo/ga=30 and time=16	30	16	0.74	0.47	1.17
Dexa vs Placebo/ga=30 and time=17	30	17	0.76	0.47	1.23
Dexa vs Placebo/ga=30 and time=18	30	18	0.79	0.48	1.31
Dexa vs Placebo/ga=30 and time=19	30	19	0.83	0.49	1.42
Dexa vs Placebo/ga=30 and time=20	30	20	0.88	0.49	1.57
Dexa vs Placebo/ga=30 and time=21	30	21	0.93	0.49	1.76
Dexa vs Placebo/ga=30 and time=22	30	22	0.99	0.49	2.01
Dexa vs Placebo/ga=30 and time=23	30	23	1.07	0.49	2.34
Dexa vs Placebo/ga=30 and time=24	30	24	1.16	0.48	2.78
Dexa vs Placebo/ga=30 and time=25	30	25	1.26	0.47	3.35
Dexa vs Placebo/ga=30 and time=26	30	26	1.38	0.46	4.10
Dexa vs Placebo/ga=30 and time=27	30	27	1.52	0.45	5.11
Dexa vs Placebo/ga=30 and time=28	30	28	1.69	0.44	6.48
Dexa vs Placebo/ga=31 and time=0	31	0	1.06	0.87	1.30
Dexa vs Placebo/ga=31 and time=1	31	1	0.99	0.84	1.17
Dexa vs Placebo/ga=31 and time=2	31	2	0.93	0.79	1.09

Dexa vs Placebo/ga=31 and time=3	31	3	0.88	0.73	1.06
Dexa vs Placebo/ga=31 and time=4	31	4	0.83	0.67	1.04
Dexa vs Placebo/ga=31 and time=5	31	5	0.80	0.62	1.03
Dexa vs Placebo/ga=31 and time=6	31	6	0.77	0.57	1.03
Dexa vs Placebo/ga=31 and time=7	31	7	0.74	0.54	1.03
Dexa vs Placebo/ga=31 and time=8	31	8	0.73	0.51	1.04
Dexa vs Placebo/ga=31 and time=9	31	9	0.71	0.49	1.04
Dexa vs Placebo/ga=31 and time=10	31	10	0.70	0.47	1.05
Dexa vs Placebo/ga=31 and time=11	31	11	0.70	0.46	1.06
Dexa vs Placebo/ga=31 and time=12	31	12	0.70	0.46	1.07
Dexa vs Placebo/ga=31 and time=13	31	13	0.71	0.46	1.09
Dexa vs Placebo/ga=31 and time=14	31	14	0.72	0.46	1.11
Dexa vs Placebo/ga=31 and time=15	31	15	0.73	0.47	1.14
Dexa vs Placebo/ga=31 and time=16	31	16	0.75	0.47	1.19
Dexa vs Placebo/ga=31 and time=17	31	17	0.77	0.48	1.25
Dexa vs Placebo/ga=31 and time=18	31	18	0.81	0.49	1.34
Dexa vs Placebo/ga=31 and time=19	31	19	0.84	0.49	1.45
Dexa vs Placebo/ga=31 and time=20	31	20	0.89	0.50	1.60
Dexa vs Placebo/ga=31 and time=21	31	21	0.94	0.50	1.79
Dexa vs Placebo/ga=31 and time=22	31	22	1.01	0.50	2.05
Dexa vs Placebo/ga=31 and time=23	31	23	1.08	0.49	2.38
Dexa vs Placebo/ga=31 and time=24	31	24	1.17	0.49	2.82
Dexa vs Placebo/ga=31 and time=25	31	25	1.27	0.48	3.39
Dexa vs Placebo/ga=31 and time=26	31	26	1.40	0.47	4.16
Dexa vs Placebo/ga=31 and time=27	31	27	1.54	0.46	5.18
Dexa vs Placebo/ga=31 and time=28	31	28	1.71	0.45	6.56
Dexa vs Placebo/ga=32 and time=0	32	0	1.08	0.86	1.35
Dexa vs Placebo/ga=32 and time=1	32	1	1.01	0.83	1.22
Dexa vs Placebo/ga=32 and time=2	32	2	0.94	0.78	1.15
Dexa vs Placebo/ga=32 and time=3	32	3	0.89	0.72	1.11
Dexa vs Placebo/ga=32 and time=4	32	4	0.85	0.66	1.09
Dexa vs Placebo/ga=32 and time=5	32	5	0.81	0.61	1.07
Dexa vs Placebo/ga=32 and time=6	32	6	0.78	0.57	1.07
Dexa vs Placebo/ga=32 and time=7	32	7	0.75	0.53	1.07
Dexa vs Placebo/ga=32 and time=8	32	8	0.74	0.51	1.07
Dexa vs Placebo/ga=32 and time=9	32	9	0.72	0.49	1.07
Dexa vs Placebo/ga=32 and time=10	32	10	0.71	0.47	1.08
Dexa vs Placebo/ga=32 and time=11	32	11	0.71	0.46	1.09
Dexa vs Placebo/ga=32 and time=12	32	12	0.71	0.46	1.10
Dexa vs Placebo/ga=32 and time=13	32	13	0.72	0.46	1.12
Dexa vs Placebo/ga=32 and time=14	32	14	0.72	0.46	1.14
Dexa vs Placebo/ga=32 and time=15	32	15	0.74	0.46	1.18
Dexa vs Placebo/ga=32 and time=16	32	16	0.76	0.47	1.22
Dexa vs Placebo/ga=32 and time=17	32	17	0.79	0.48	1.29
Dexa vs Placebo/ga=32 and time=18	32	18	0.82	0.49	1.37
Dexa vs Placebo/ga=32 and time=19	32	19	0.86	0.49	1.48
Dexa vs Placebo/ga=32 and time=20	32	20	0.90	0.50	1.63
Dexa vs Placebo/ga=32 and time=21	32	21	0.96	0.50	1.83
Dexa vs Placebo/ga=32 and time=22	32	22	1.02	0.50	2.09
Dexa vs Placebo/ga=32 and time=23	32	23	1.10	0.50	2.43
Dexa vs Placebo/ga=32 and time=24	32	24	1.19	0.49	2.87
Dexa vs Placebo/ga=32 and time=25	32	25	1.29	0.48	3.45

Dexa vs Placebo/ga=32 and time=26	32	26	1.42	0.47	4.23
Dexa vs Placebo/ga=32 and time=27	32	27	1.56	0.46	5.26
Dexa vs Placebo/ga=32 and time=28	32	28	1.73	0.45	6.66
Dexa vs Placebo/ga=33 and time=0	33	0	1.09	0.84	1.42
Dexa vs Placebo/ga=33 and time=1	33	1	1.02	0.80	1.30
Dexa vs Placebo/ga=33 and time=2	33	2	0.96	0.75	1.22
Dexa vs Placebo/ga=33 and time=3	33	3	0.90	0.70	1.17
Dexa vs Placebo/ga=33 and time=4	33	4	0.86	0.64	1.14
Dexa vs Placebo/ga=33 and time=5	33	5	0.82	0.60	1.13
Dexa vs Placebo/ga=33 and time=6	33	6	0.79	0.56	1.12
Dexa vs Placebo/ga=33 and time=7	33	7	0.77	0.52	1.12
Dexa vs Placebo/ga=33 and time=8	33	8	0.75	0.50	1.12
Dexa vs Placebo/ga=33 and time=9	33	9	0.73	0.48	1.12
Dexa vs Placebo/ga=33 and time=10	33	10	0.72	0.47	1.12
Dexa vs Placebo/ga=33 and time=11	33	11	0.72	0.46	1.13
Dexa vs Placebo/ga=33 and time=12	33	12	0.72	0.45	1.14
Dexa vs Placebo/ga=33 and time=13	33	13	0.73	0.45	1.16
Dexa vs Placebo/ga=33 and time=14	33	14	0.74	0.45	1.19
Dexa vs Placebo/ga=33 and time=15	33	15	0.75	0.46	1.22
Dexa vs Placebo/ga=33 and time=16	33	16	0.77	0.47	1.27
Dexa vs Placebo/ga=33 and time=17	33	17	0.80	0.48	1.33
Dexa vs Placebo/ga=33 and time=18	33	18	0.83	0.48	1.42
Dexa vs Placebo/ga=33 and time=19	33	19	0.87	0.49	1.53
Dexa vs Placebo/ga=33 and time=20	33	20	0.91	0.50	1.68
Dexa vs Placebo/ga=33 and time=21	33	21	0.97	0.50	1.88
Dexa vs Placebo/ga=33 and time=22	33	22	1.04	0.50	2.14
Dexa vs Placebo/ga=33 and time=23	33	23	1.11	0.50	2.49
Dexa vs Placebo/ga=33 and time=24	33	24	1.20	0.49	2.94
Dexa vs Placebo/ga=33 and time=25	33	25	1.31	0.49	3.53
Dexa vs Placebo/ga=33 and time=26	33	26	1.44	0.48	4.31
Dexa vs Placebo/ga=33 and time=27	33	27	1.58	0.47	5.36
Dexa vs Placebo/ga=33 and time=28	33	28	1.76	0.46	6.78

Supplementary Table 1d. Severe respiratory distress at 24h (0-28d)

Label	Gestational age at first dose, weeks	Administration-to-birth interval, days	RR	Lower 95%CL	Upper 95%CL
Dexa vs Placebo/ga=26 and time=0	26	0	0.25	0.07	0.85
Dexa vs Placebo/ga=26 and time=1	26	1	0.22	0.07	0.70
Dexa vs Placebo/ga=26 and time=2	26	2	0.20	0.06	0.62
Dexa vs Placebo/ga=26 and time=3	26	3	0.18	0.06	0.57
Dexa vs Placebo/ga=26 and time=4	26	4	0.16	0.05	0.55
Dexa vs Placebo/ga=26 and time=5	26	5	0.15	0.04	0.54
Dexa vs Placebo/ga=26 and time=6	26	6	0.14	0.04	0.54
Dexa vs Placebo/ga=26 and time=7	26	7	0.13	0.03	0.54
Dexa vs Placebo/ga=26 and time=8	26	8	0.13	0.03	0.55
Dexa vs Placebo/ga=26 and time=9	26	9	0.12	0.03	0.55
Dexa vs Placebo/ga=26 and time=10	26	10	0.12	0.03	0.56
Dexa vs Placebo/ga=26 and time=11	26	11	0.12	0.02	0.57
Dexa vs Placebo/ga=26 and time=12	26	12	0.12	0.02	0.60
Dexa vs Placebo/ga=26 and time=13	26	13	0.12	0.02	0.63
Dexa vs Placebo/ga=26 and time=14	26	14	0.12	0.02	0.70
Dexa vs Placebo/ga=26 and time=15	26	15	0.12	0.02	0.79
Dexa vs Placebo/ga=26 and time=16	26	16	0.13	0.02	0.94
Dexa vs Placebo/ga=26 and time=17	26	17	0.13	0.02	1.18
Dexa vs Placebo/ga=26 and time=18	26	18	0.14	0.01	1.56
Dexa vs Placebo/ga=26 and time=19	26	19	0.15	0.01	2.19
Dexa vs Placebo/ga=26 and time=20	26	20	0.17	0.01	3.23
Dexa vs Placebo/ga=26 and time=21	26	21	0.18	0.01	5.05
Dexa vs Placebo/ga=26 and time=22	26	22	0.20	0.00	8.32
Dexa vs Placebo/ga=26 and time=23	26	23	0.22	0.00	14.43
Dexa vs Placebo/ga=26 and time=24	26	24	0.25	0.00	26.36
Dexa vs Placebo/ga=26 and time=25	26	25	0.29	0.00	50.62
Dexa vs Placebo/ga=26 and time=26	26	26	0.33	0.00	102.10
Dexa vs Placebo/ga=26 and time=27	26	27	0.38	0.00	216.11
Dexa vs Placebo/ga=26 and time=28	26	28	0.45	0.00	479.76
Dexa vs Placebo/ga=27 and time=0	27	0	0.31	0.11	0.89
Dexa vs Placebo/ga=27 and time=1	27	1	0.27	0.10	0.72
Dexa vs Placebo/ga=27 and time=2	27	2	0.25	0.09	0.64
Dexa vs Placebo/ga=27 and time=3	27	3	0.22	0.08	0.60
Dexa vs Placebo/ga=27 and time=4	27	4	0.20	0.07	0.58
Dexa vs Placebo/ga=27 and time=5	27	5	0.19	0.06	0.58
Dexa vs Placebo/ga=27 and time=6	27	6	0.18	0.05	0.58
Dexa vs Placebo/ga=27 and time=7	27	7	0.17	0.05	0.59
Dexa vs Placebo/ga=27 and time=8	27	8	0.16	0.04	0.60
Dexa vs Placebo/ga=27 and time=9	27	9	0.15	0.04	0.60
Dexa vs Placebo/ga=27 and time=10	27	10	0.15	0.04	0.62
Dexa vs Placebo/ga=27 and time=11	27	11	0.15	0.03	0.63
Dexa vs Placebo/ga=27 and time=12	27	12	0.15	0.03	0.66
Dexa vs Placebo/ga=27 and time=13	27	13	0.15	0.03	0.71
Dexa vs Placebo/ga=27 and time=14	27	14	0.15	0.03	0.78
Dexa vs Placebo/ga=27 and time=15	27	15	0.16	0.03	0.89
Dexa vs Placebo/ga=27 and time=16	27	16	0.16	0.02	1.07

Dexa vs Placebo/ga=27 and time=17	27	17	0.17	0.02	1.35
Dexa vs Placebo/ga=27 and time=18	27	18	0.18	0.02	1.80
Dexa vs Placebo/ga=27 and time=19	27	19	0.19	0.01	2.54
Dexa vs Placebo/ga=27 and time=20	27	20	0.21	0.01	3.78
Dexa vs Placebo/ga=27 and time=21	27	21	0.23	0.01	5.95
Dexa vs Placebo/ga=27 and time=22	27	22	0.25	0.01	9.85
Dexa vs Placebo/ga=27 and time=23	27	23	0.28	0.00	17.19
Dexa vs Placebo/ga=27 and time=24	27	24	0.32	0.00	31.54
Dexa vs Placebo/ga=27 and time=25	27	25	0.36	0.00	60.80
Dexa vs Placebo/ga=27 and time=26	27	26	0.41	0.00	123.06
Dexa vs Placebo/ga=27 and time=27	27	27	0.48	0.00	261.25
Dexa vs Placebo/ga=27 and time=28	27	28	0.57	0.00	581.47
Dexa vs Placebo/ga=28 and time=0	28	0	0.39	0.16	0.95
Dexa vs Placebo/ga=28 and time=1	28	1	0.34	0.16	0.76
Dexa vs Placebo/ga=28 and time=2	28	2	0.31	0.14	0.67
Dexa vs Placebo/ga=28 and time=3	28	3	0.28	0.12	0.63
Dexa vs Placebo/ga=28 and time=4	28	4	0.26	0.11	0.62
Dexa vs Placebo/ga=28 and time=5	28	5	0.24	0.09	0.63
Dexa vs Placebo/ga=28 and time=6	28	6	0.22	0.08	0.64
Dexa vs Placebo/ga=28 and time=7	28	7	0.21	0.07	0.65
Dexa vs Placebo/ga=28 and time=8	28	8	0.20	0.06	0.67
Dexa vs Placebo/ga=28 and time=9	28	9	0.19	0.06	0.68
Dexa vs Placebo/ga=28 and time=10	28	10	0.19	0.05	0.70
Dexa vs Placebo/ga=28 and time=11	28	11	0.19	0.05	0.72
Dexa vs Placebo/ga=28 and time=12	28	12	0.19	0.05	0.75
Dexa vs Placebo/ga=28 and time=13	28	13	0.19	0.04	0.81
Dexa vs Placebo/ga=28 and time=14	28	14	0.19	0.04	0.89
Dexa vs Placebo/ga=28 and time=15	28	15	0.20	0.04	1.03
Dexa vs Placebo/ga=28 and time=16	28	16	0.20	0.03	1.24
Dexa vs Placebo/ga=28 and time=17	28	17	0.21	0.03	1.58
Dexa vs Placebo/ga=28 and time=18	28	18	0.23	0.02	2.12
Dexa vs Placebo/ga=28 and time=19	28	19	0.24	0.02	3.00
Dexa vs Placebo/ga=28 and time=20	28	20	0.26	0.02	4.50
Dexa vs Placebo/ga=28 and time=21	28	21	0.29	0.01	7.10
Dexa vs Placebo/ga=28 and time=22	28	22	0.32	0.01	11.82
Dexa vs Placebo/ga=28 and time=23	28	23	0.35	0.01	20.70
Dexa vs Placebo/ga=28 and time=24	28	24	0.40	0.00	38.11
Dexa vs Placebo/ga=28 and time=25	28	25	0.45	0.00	73.70
Dexa vs Placebo/ga=28 and time=26	28	26	0.52	0.00	149.53
Dexa vs Placebo/ga=28 and time=27	28	27	0.61	0.00	318.15
Dexa vs Placebo/ga=28 and time=28	28	28	0.71	0.00	709.51
Dexa vs Placebo/ga=29 and time=0	29	0	0.48	0.23	1.03
Dexa vs Placebo/ga=29 and time=1	29	1	0.43	0.23	0.81
Dexa vs Placebo/ga=29 and time=2	29	2	0.39	0.21	0.71
Dexa vs Placebo/ga=29 and time=3	29	3	0.35	0.18	0.69
Dexa vs Placebo/ga=29 and time=4	29	4	0.32	0.15	0.69
Dexa vs Placebo/ga=29 and time=5	29	5	0.30	0.13	0.71
Dexa vs Placebo/ga=29 and time=6	29	6	0.28	0.11	0.73
Dexa vs Placebo/ga=29 and time=7	29	7	0.26	0.09	0.75
Dexa vs Placebo/ga=29 and time=8	29	8	0.25	0.08	0.77
Dexa vs Placebo/ga=29 and time=9	29	9	0.24	0.07	0.79
Dexa vs Placebo/ga=29 and time=10	29	10	0.24	0.07	0.81

Dexa vs Placebo/ga=29 and time=11	29	11	0.23	0.07	0.84
Dexa vs Placebo/ga=29 and time=12	29	12	0.23	0.06	0.88
Dexa vs Placebo/ga=29 and time=13	29	13	0.23	0.06	0.95
Dexa vs Placebo/ga=29 and time=14	29	14	0.24	0.05	1.05
Dexa vs Placebo/ga=29 and time=15	29	15	0.24	0.05	1.21
Dexa vs Placebo/ga=29 and time=16	29	16	0.25	0.04	1.47
Dexa vs Placebo/ga=29 and time=17	29	17	0.27	0.04	1.88
Dexa vs Placebo/ga=29 and time=18	29	18	0.28	0.03	2.54
Dexa vs Placebo/ga=29 and time=19	29	19	0.30	0.03	3.61
Dexa vs Placebo/ga=29 and time=20	29	20	0.33	0.02	5.43
Dexa vs Placebo/ga=29 and time=21	29	21	0.36	0.01	8.60
Dexa vs Placebo/ga=29 and time=22	29	22	0.40	0.01	14.36
Dexa vs Placebo/ga=29 and time=23	29	23	0.44	0.01	25.22
Dexa vs Placebo/ga=29 and time=24	29	24	0.50	0.01	46.53
Dexa vs Placebo/ga=29 and time=25	29	25	0.57	0.00	90.14
Dexa vs Placebo/ga=29 and time=26	29	26	0.65	0.00	183.20
Dexa vs Placebo/ga=29 and time=27	29	27	0.76	0.00	390.37
Dexa vs Placebo/ga=29 and time=28	29	28	0.89	0.00	871.66
Dexa vs Placebo/ga=30 and time=0	30	0	0.61	0.31	1.18
Dexa vs Placebo/ga=30 and time=1	30	1	0.54	0.32	0.91
Dexa vs Placebo/ga=30 and time=2	30	2	0.49	0.29	0.80
Dexa vs Placebo/ga=30 and time=3	30	3	0.44	0.25	0.78
Dexa vs Placebo/ga=30 and time=4	30	4	0.40	0.20	0.80
Dexa vs Placebo/ga=30 and time=5	30	5	0.37	0.17	0.83
Dexa vs Placebo/ga=30 and time=6	30	6	0.35	0.14	0.87
Dexa vs Placebo/ga=30 and time=7	30	7	0.33	0.12	0.90
Dexa vs Placebo/ga=30 and time=8	30	8	0.32	0.11	0.92
Dexa vs Placebo/ga=30 and time=9	30	9	0.31	0.10	0.95
Dexa vs Placebo/ga=30 and time=10	30	10	0.30	0.09	0.98
Dexa vs Placebo/ga=30 and time=11	30	11	0.29	0.08	1.01
Dexa vs Placebo/ga=30 and time=12	30	12	0.29	0.08	1.07
Dexa vs Placebo/ga=30 and time=13	30	13	0.29	0.08	1.15
Dexa vs Placebo/ga=30 and time=14	30	14	0.30	0.07	1.27
Dexa vs Placebo/ga=30 and time=15	30	15	0.31	0.06	1.48
Dexa vs Placebo/ga=30 and time=16	30	16	0.32	0.06	1.79
Dexa vs Placebo/ga=30 and time=17	30	17	0.33	0.05	2.29
Dexa vs Placebo/ga=30 and time=18	30	18	0.35	0.04	3.10
Dexa vs Placebo/ga=30 and time=19	30	19	0.38	0.03	4.42
Dexa vs Placebo/ga=30 and time=20	30	20	0.41	0.03	6.66
Dexa vs Placebo/ga=30 and time=21	30	21	0.45	0.02	10.57
Dexa vs Placebo/ga=30 and time=22	30	22	0.50	0.01	17.67
Dexa vs Placebo/ga=30 and time=23	30	23	0.55	0.01	31.07
Dexa vs Placebo/ga=30 and time=24	30	24	0.62	0.01	57.40
Dexa vs Placebo/ga=30 and time=25	30	25	0.71	0.00	111.29
Dexa vs Placebo/ga=30 and time=26	30	26	0.82	0.00	226.36
Dexa vs Placebo/ga=30 and time=27	30	27	0.95	0.00	482.63
Dexa vs Placebo/ga=30 and time=28	30	28	1.12	0.00	1078.25
Dexa vs Placebo/ga=31 and time=0	31	0	0.76	0.41	1.43
Dexa vs Placebo/ga=31 and time=1	31	1	0.68	0.41	1.11
Dexa vs Placebo/ga=31 and time=2	31	2	0.61	0.38	0.98
Dexa vs Placebo/ga=31 and time=3	31	3	0.55	0.32	0.97
Dexa vs Placebo/ga=31 and time=4	31	4	0.51	0.26	0.99

Dexa vs Placebo/ga=31 and time=5	31	5	0.47	0.21	1.04
Dexa vs Placebo/ga=31 and time=6	31	6	0.44	0.18	1.08
Dexa vs Placebo/ga=31 and time=7	31	7	0.42	0.15	1.12
Dexa vs Placebo/ga=31 and time=8	31	8	0.40	0.14	1.16
Dexa vs Placebo/ga=31 and time=9	31	9	0.38	0.12	1.19
Dexa vs Placebo/ga=31 and time=10	31	10	0.37	0.11	1.23
Dexa vs Placebo/ga=31 and time=11	31	11	0.37	0.11	1.27
Dexa vs Placebo/ga=31 and time=12	31	12	0.37	0.10	1.34
Dexa vs Placebo/ga=31 and time=13	31	13	0.37	0.09	1.44
Dexa vs Placebo/ga=31 and time=14	31	14	0.38	0.09	1.60
Dexa vs Placebo/ga=31 and time=15	31	15	0.39	0.08	1.85
Dexa vs Placebo/ga=31 and time=16	31	16	0.40	0.07	2.24
Dexa vs Placebo/ga=31 and time=17	31	17	0.42	0.06	2.87
Dexa vs Placebo/ga=31 and time=18	31	18	0.44	0.05	3.87
Dexa vs Placebo/ga=31 and time=19	31	19	0.48	0.04	5.52
Dexa vs Placebo/ga=31 and time=20	31	20	0.52	0.03	8.31
Dexa vs Placebo/ga=31 and time=21	31	21	0.56	0.02	13.19
Dexa vs Placebo/ga=31 and time=22	31	22	0.62	0.02	22.05
Dexa vs Placebo/ga=31 and time=23	31	23	0.70	0.01	38.75
Dexa vs Placebo/ga=31 and time=24	31	24	0.78	0.01	71.55
Dexa vs Placebo/ga=31 and time=25	31	25	0.89	0.01	138.70
Dexa vs Placebo/ga=31 and time=26	31	26	1.03	0.00	282.05
Dexa vs Placebo/ga=31 and time=27	31	27	1.20	0.00	601.27
Dexa vs Placebo/ga=31 and time=28	31	28	1.41	0.00	1343.08
Dexa vs Placebo/ga=32 and time=0	32	0	0.96	0.49	1.88
Dexa vs Placebo/ga=32 and time=1	32	1	0.85	0.49	1.48
Dexa vs Placebo/ga=32 and time=2	32	2	0.76	0.44	1.32
Dexa vs Placebo/ga=32 and time=3	32	3	0.69	0.37	1.29
Dexa vs Placebo/ga=32 and time=4	32	4	0.64	0.31	1.32
Dexa vs Placebo/ga=32 and time=5	32	5	0.59	0.25	1.37
Dexa vs Placebo/ga=32 and time=6	32	6	0.55	0.21	1.42
Dexa vs Placebo/ga=32 and time=7	32	7	0.52	0.18	1.47
Dexa vs Placebo/ga=32 and time=8	32	8	0.50	0.16	1.52
Dexa vs Placebo/ga=32 and time=9	32	9	0.48	0.15	1.56
Dexa vs Placebo/ga=32 and time=10	32	10	0.47	0.14	1.60
Dexa vs Placebo/ga=32 and time=11	32	11	0.46	0.13	1.66
Dexa vs Placebo/ga=32 and time=12	32	12	0.46	0.12	1.74
Dexa vs Placebo/ga=32 and time=13	32	13	0.46	0.11	1.87
Dexa vs Placebo/ga=32 and time=14	32	14	0.47	0.11	2.07
Dexa vs Placebo/ga=32 and time=15	32	15	0.48	0.10	2.39
Dexa vs Placebo/ga=32 and time=16	32	16	0.50	0.09	2.89
Dexa vs Placebo/ga=32 and time=17	32	17	0.53	0.08	3.68
Dexa vs Placebo/ga=32 and time=18	32	18	0.56	0.06	4.95
Dexa vs Placebo/ga=32 and time=19	32	19	0.60	0.05	7.03
Dexa vs Placebo/ga=32 and time=20	32	20	0.65	0.04	10.56
Dexa vs Placebo/ga=32 and time=21	32	21	0.71	0.03	16.71
Dexa vs Placebo/ga=32 and time=22	32	22	0.78	0.02	27.87
Dexa vs Placebo/ga=32 and time=23	32	23	0.87	0.02	48.89
Dexa vs Placebo/ga=32 and time=24	32	24	0.98	0.01	90.14
Dexa vs Placebo/ga=32 and time=25	32	25	1.12	0.01	174.50
Dexa vs Placebo/ga=32 and time=26	32	26	1.29	0.00	354.44
Dexa vs Placebo/ga=32 and time=27	32	27	1.50	0.00	754.83

Dexa vs Placebo/ga=32 and time=28	32	28	1.77	0.00	1684.61
Dexa vs Placebo/ga=33 and time=0	33	0	1.20	0.55	2.61
Dexa vs Placebo/ga=33 and time=1	33	1	1.07	0.54	2.11
Dexa vs Placebo/ga=33 and time=2	33	2	0.96	0.49	1.89
Dexa vs Placebo/ga=33 and time=3	33	3	0.87	0.41	1.84
Dexa vs Placebo/ga=33 and time=4	33	4	0.80	0.34	1.85
Dexa vs Placebo/ga=33 and time=5	33	5	0.74	0.29	1.90
Dexa vs Placebo/ga=33 and time=6	33	6	0.69	0.24	1.96
Dexa vs Placebo/ga=33 and time=7	33	7	0.65	0.21	2.01
Dexa vs Placebo/ga=33 and time=8	33	8	0.62	0.19	2.06
Dexa vs Placebo/ga=33 and time=9	33	9	0.60	0.17	2.11
Dexa vs Placebo/ga=33 and time=10	33	10	0.59	0.16	2.17
Dexa vs Placebo/ga=33 and time=11	33	11	0.58	0.15	2.24
Dexa vs Placebo/ga=33 and time=12	33	12	0.58	0.14	2.34
Dexa vs Placebo/ga=33 and time=13	33	13	0.58	0.13	2.50
Dexa vs Placebo/ga=33 and time=14	33	14	0.59	0.13	2.76
Dexa vs Placebo/ga=33 and time=15	33	15	0.61	0.12	3.17
Dexa vs Placebo/ga=33 and time=16	33	16	0.63	0.10	3.81
Dexa vs Placebo/ga=33 and time=17	33	17	0.66	0.09	4.83
Dexa vs Placebo/ga=33 and time=18	33	18	0.70	0.08	6.46
Dexa vs Placebo/ga=33 and time=19	33	19	0.75	0.06	9.13
Dexa vs Placebo/ga=33 and time=20	33	20	0.81	0.05	13.64
Dexa vs Placebo/ga=33 and time=21	33	21	0.89	0.04	21.49
Dexa vs Placebo/ga=33 and time=22	33	22	0.98	0.03	35.70
Dexa vs Placebo/ga=33 and time=23	33	23	1.09	0.02	62.42
Dexa vs Placebo/ga=33 and time=24	33	24	1.23	0.01	114.76
Dexa vs Placebo/ga=33 and time=25	33	25	1.41	0.01	221.63
Dexa vs Placebo/ga=33 and time=26	33	26	1.62	0.01	449.20
Dexa vs Placebo/ga=33 and time=27	33	27	1.89	0.00	954.89
Dexa vs Placebo/ga=33 and time=28	33	28	2.22	0.00	2127.72

Supplementary able 1e. Severe respiratory distress at 7 days (0-28d)

Label	Gestational age at first dose, weeks	Administration-to-birth interval, days	RR	Lower 95%CL	Upper 95%CL
Dexa vs Placebo/ga=26 and time=0	26	0	0.73	0.37	1.44
Dexa vs Placebo/ga=26 and time=1	26	1	0.66	0.35	1.25
Dexa vs Placebo/ga=26 and time=2	26	2	0.60	0.32	1.14
Dexa vs Placebo/ga=26 and time=3	26	3	0.55	0.28	1.07
Dexa vs Placebo/ga=26 and time=4	26	4	0.51	0.25	1.03
Dexa vs Placebo/ga=26 and time=5	26	5	0.47	0.22	1.00
Dexa vs Placebo/ga=26 and time=6	26	6	0.44	0.20	0.99
Dexa vs Placebo/ga=26 and time=7	26	7	0.42	0.18	0.97
Dexa vs Placebo/ga=26 and time=8	26	8	0.40	0.16	0.96
Dexa vs Placebo/ga=26 and time=9	26	9	0.38	0.15	0.95
Dexa vs Placebo/ga=26 and time=10	26	10	0.36	0.14	0.94
Dexa vs Placebo/ga=26 and time=11	26	11	0.35	0.13	0.94
Dexa vs Placebo/ga=26 and time=12	26	12	0.34	0.12	0.94
Dexa vs Placebo/ga=26 and time=13	26	13	0.34	0.12	0.95
Dexa vs Placebo/ga=26 and time=14	26	14	0.33	0.11	0.97
Dexa vs Placebo/ga=26 and time=15	26	15	0.33	0.11	1.01
Dexa vs Placebo/ga=26 and time=16	26	16	0.33	0.10	1.08
Dexa vs Placebo/ga=26 and time=17	26	17	0.33	0.09	1.18
Dexa vs Placebo/ga=26 and time=18	26	18	0.34	0.09	1.32
Dexa vs Placebo/ga=26 and time=19	26	19	0.34	0.08	1.52
Dexa vs Placebo/ga=26 and time=20	26	20	0.35	0.07	1.81
Dexa vs Placebo/ga=26 and time=21	26	21	0.36	0.06	2.23
Dexa vs Placebo/ga=26 and time=22	26	22	0.38	0.05	2.82
Dexa vs Placebo/ga=26 and time=23	26	23	0.40	0.04	3.69
Dexa vs Placebo/ga=26 and time=24	26	24	0.42	0.04	4.97
Dexa vs Placebo/ga=26 and time=25	26	25	0.45	0.03	6.90
Dexa vs Placebo/ga=26 and time=26	26	26	0.48	0.02	9.86
Dexa vs Placebo/ga=26 and time=27	26	27	0.51	0.02	14.49
Dexa vs Placebo/ga=26 and time=28	26	28	0.56	0.01	21.92
Dexa vs Placebo/ga=27 and time=0	27	0	0.78	0.43	1.39
Dexa vs Placebo/ga=27 and time=1	27	1	0.70	0.41	1.20
Dexa vs Placebo/ga=27 and time=2	27	2	0.64	0.38	1.09
Dexa vs Placebo/ga=27 and time=3	27	3	0.59	0.33	1.03
Dexa vs Placebo/ga=27 and time=4	27	4	0.54	0.29	1.00
Dexa vs Placebo/ga=27 and time=5	27	5	0.50	0.26	0.98
Dexa vs Placebo/ga=27 and time=6	27	6	0.47	0.23	0.97
Dexa vs Placebo/ga=27 and time=7	27	7	0.44	0.20	0.97
Dexa vs Placebo/ga=27 and time=8	27	8	0.42	0.19	0.96
Dexa vs Placebo/ga=27 and time=9	27	9	0.40	0.17	0.95
Dexa vs Placebo/ga=27 and time=10	27	10	0.39	0.16	0.95
Dexa vs Placebo/ga=27 and time=11	27	11	0.37	0.15	0.94
Dexa vs Placebo/ga=27 and time=12	27	12	0.36	0.14	0.95
Dexa vs Placebo/ga=27 and time=13	27	13	0.36	0.13	0.96
Dexa vs Placebo/ga=27 and time=14	27	14	0.35	0.13	0.98
Dexa vs Placebo/ga=27 and time=15	27	15	0.35	0.12	1.02

Dexa vs Placebo/ga=27 and time=16	27	16	0.35	0.11	1.09
Dexa vs Placebo/ga=27 and time=17	27	17	0.35	0.10	1.19
Dexa vs Placebo/ga=27 and time=18	27	18	0.36	0.10	1.34
Dexa vs Placebo/ga=27 and time=19	27	19	0.37	0.09	1.55
Dexa vs Placebo/ga=27 and time=20	27	20	0.38	0.08	1.86
Dexa vs Placebo/ga=27 and time=21	27	21	0.39	0.07	2.29
Dexa vs Placebo/ga=27 and time=22	27	22	0.40	0.06	2.91
Dexa vs Placebo/ga=27 and time=23	27	23	0.42	0.05	3.81
Dexa vs Placebo/ga=27 and time=24	27	24	0.45	0.04	5.15
Dexa vs Placebo/ga=27 and time=25	27	25	0.48	0.03	7.17
Dexa vs Placebo/ga=27 and time=26	27	26	0.51	0.03	10.26
Dexa vs Placebo/ga=27 and time=27	27	27	0.55	0.02	15.12
Dexa vs Placebo/ga=27 and time=28	27	28	0.59	0.02	22.89
Dexa vs Placebo/ga=28 and time=0	28	0	0.83	0.51	1.35
Dexa vs Placebo/ga=28 and time=1	28	1	0.75	0.49	1.16
Dexa vs Placebo/ga=28 and time=2	28	2	0.68	0.44	1.05
Dexa vs Placebo/ga=28 and time=3	28	3	0.63	0.39	1.00
Dexa vs Placebo/ga=28 and time=4	28	4	0.58	0.34	0.99
Dexa vs Placebo/ga=28 and time=5	28	5	0.54	0.30	0.98
Dexa vs Placebo/ga=28 and time=6	28	6	0.50	0.26	0.97
Dexa vs Placebo/ga=28 and time=7	28	7	0.47	0.23	0.97
Dexa vs Placebo/ga=28 and time=8	28	8	0.45	0.21	0.97
Dexa vs Placebo/ga=28 and time=9	28	9	0.43	0.19	0.96
Dexa vs Placebo/ga=28 and time=10	28	10	0.41	0.18	0.96
Dexa vs Placebo/ga=28 and time=11	28	11	0.40	0.17	0.96
Dexa vs Placebo/ga=28 and time=12	28	12	0.39	0.16	0.96
Dexa vs Placebo/ga=28 and time=13	28	13	0.38	0.15	0.98
Dexa vs Placebo/ga=28 and time=14	28	14	0.38	0.14	1.00
Dexa vs Placebo/ga=28 and time=15	28	15	0.37	0.13	1.05
Dexa vs Placebo/ga=28 and time=16	28	16	0.37	0.13	1.12
Dexa vs Placebo/ga=28 and time=17	28	17	0.38	0.12	1.22
Dexa vs Placebo/ga=28 and time=18	28	18	0.38	0.11	1.38
Dexa vs Placebo/ga=28 and time=19	28	19	0.39	0.10	1.60
Dexa vs Placebo/ga=28 and time=20	28	20	0.40	0.08	1.92
Dexa vs Placebo/ga=28 and time=21	28	21	0.41	0.07	2.37
Dexa vs Placebo/ga=28 and time=22	28	22	0.43	0.06	3.02
Dexa vs Placebo/ga=28 and time=23	28	23	0.45	0.05	3.97
Dexa vs Placebo/ga=28 and time=24	28	24	0.48	0.04	5.37
Dexa vs Placebo/ga=28 and time=25	28	25	0.51	0.03	7.49
Dexa vs Placebo/ga=28 and time=26	28	26	0.54	0.03	10.74
Dexa vs Placebo/ga=28 and time=27	28	27	0.58	0.02	15.83
Dexa vs Placebo/ga=28 and time=28	28	28	0.63	0.02	24.01
Dexa vs Placebo/ga=29 and time=0	29	0	0.88	0.59	1.33
Dexa vs Placebo/ga=29 and time=1	29	1	0.80	0.57	1.13
Dexa vs Placebo/ga=29 and time=2	29	2	0.73	0.52	1.03
Dexa vs Placebo/ga=29 and time=3	29	3	0.67	0.45	1.00
Dexa vs Placebo/ga=29 and time=4	29	4	0.62	0.39	0.99
Dexa vs Placebo/ga=29 and time=5	29	5	0.57	0.33	0.99
Dexa vs Placebo/ga=29 and time=6	29	6	0.54	0.29	0.99
Dexa vs Placebo/ga=29 and time=7	29	7	0.51	0.26	0.99
Dexa vs Placebo/ga=29 and time=8	29	8	0.48	0.23	0.99
Dexa vs Placebo/ga=29 and time=9	29	9	0.46	0.21	0.99

Dexa vs Placebo/ga=29 and time=10	29	10	0.44	0.20	0.99
Dexa vs Placebo/ga=29 and time=11	29	11	0.43	0.18	0.99
Dexa vs Placebo/ga=29 and time=12	29	12	0.42	0.17	0.99
Dexa vs Placebo/ga=29 and time=13	29	13	0.41	0.16	1.01
Dexa vs Placebo/ga=29 and time=14	29	14	0.40	0.16	1.04
Dexa vs Placebo/ga=29 and time=15	29	15	0.40	0.15	1.08
Dexa vs Placebo/ga=29 and time=16	29	16	0.40	0.14	1.16
Dexa vs Placebo/ga=29 and time=17	29	17	0.40	0.13	1.27
Dexa vs Placebo/ga=29 and time=18	29	18	0.41	0.12	1.43
Dexa vs Placebo/ga=29 and time=19	29	19	0.42	0.10	1.67
Dexa vs Placebo/ga=29 and time=20	29	20	0.43	0.09	2.00
Dexa vs Placebo/ga=29 and time=21	29	21	0.44	0.08	2.48
Dexa vs Placebo/ga=29 and time=22	29	22	0.46	0.07	3.16
Dexa vs Placebo/ga=29 and time=23	29	23	0.48	0.06	4.16
Dexa vs Placebo/ga=29 and time=24	29	24	0.51	0.05	5.64
Dexa vs Placebo/ga=29 and time=25	29	25	0.54	0.04	7.86
Dexa vs Placebo/ga=29 and time=26	29	26	0.58	0.03	11.29
Dexa vs Placebo/ga=29 and time=27	29	27	0.62	0.02	16.66
Dexa vs Placebo/ga=29 and time=28	29	28	0.68	0.02	25.28
Dexa vs Placebo/ga=30 and time=0	30	0	0.94	0.66	1.35
Dexa vs Placebo/ga=30 and time=1	30	1	0.85	0.64	1.13
Dexa vs Placebo/ga=30 and time=2	30	2	0.78	0.58	1.04
Dexa vs Placebo/ga=30 and time=3	30	3	0.71	0.50	1.02
Dexa vs Placebo/ga=30 and time=4	30	4	0.66	0.43	1.02
Dexa vs Placebo/ga=30 and time=5	30	5	0.61	0.37	1.02
Dexa vs Placebo/ga=30 and time=6	30	6	0.57	0.32	1.03
Dexa vs Placebo/ga=30 and time=7	30	7	0.54	0.28	1.04
Dexa vs Placebo/ga=30 and time=8	30	8	0.51	0.25	1.04
Dexa vs Placebo/ga=30 and time=9	30	9	0.49	0.23	1.04
Dexa vs Placebo/ga=30 and time=10	30	10	0.47	0.21	1.04
Dexa vs Placebo/ga=30 and time=11	30	11	0.46	0.20	1.04
Dexa vs Placebo/ga=30 and time=12	30	12	0.44	0.19	1.04
Dexa vs Placebo/ga=30 and time=13	30	13	0.44	0.18	1.06
Dexa vs Placebo/ga=30 and time=14	30	14	0.43	0.17	1.09
Dexa vs Placebo/ga=30 and time=15	30	15	0.43	0.16	1.14
Dexa vs Placebo/ga=30 and time=16	30	16	0.43	0.15	1.22
Dexa vs Placebo/ga=30 and time=17	30	17	0.43	0.14	1.34
Dexa vs Placebo/ga=30 and time=18	30	18	0.44	0.13	1.51
Dexa vs Placebo/ga=30 and time=19	30	19	0.45	0.11	1.76
Dexa vs Placebo/ga=30 and time=20	30	20	0.46	0.10	2.11
Dexa vs Placebo/ga=30 and time=21	30	21	0.47	0.09	2.61
Dexa vs Placebo/ga=30 and time=22	30	22	0.49	0.07	3.33
Dexa vs Placebo/ga=30 and time=23	30	23	0.52	0.06	4.39
Dexa vs Placebo/ga=30 and time=24	30	24	0.54	0.05	5.95
Dexa vs Placebo/ga=30 and time=25	30	25	0.58	0.04	8.30
Dexa vs Placebo/ga=30 and time=26	30	26	0.62	0.03	11.92
Dexa vs Placebo/ga=30 and time=27	30	27	0.67	0.03	17.61
Dexa vs Placebo/ga=30 and time=28	30	28	0.72	0.02	26.72
Dexa vs Placebo/ga=31 and time=0	31	0	1.01	0.72	1.41
Dexa vs Placebo/ga=31 and time=1	31	1	0.91	0.70	1.19
Dexa vs Placebo/ga=31 and time=2	31	2	0.83	0.63	1.10
Dexa vs Placebo/ga=31 and time=3	31	3	0.76	0.54	1.08

Dexa vs Placebo/ga=31 and time=4	31	4	0.70	0.46	1.08
Dexa vs Placebo/ga=31 and time=5	31	5	0.66	0.39	1.09
Dexa vs Placebo/ga=31 and time=6	31	6	0.61	0.34	1.10
Dexa vs Placebo/ga=31 and time=7	31	7	0.58	0.30	1.11
Dexa vs Placebo/ga=31 and time=8	31	8	0.55	0.27	1.11
Dexa vs Placebo/ga=31 and time=9	31	9	0.52	0.25	1.11
Dexa vs Placebo/ga=31 and time=10	31	10	0.50	0.23	1.11
Dexa vs Placebo/ga=31 and time=11	31	11	0.49	0.21	1.11
Dexa vs Placebo/ga=31 and time=12	31	12	0.47	0.20	1.11
Dexa vs Placebo/ga=31 and time=13	31	13	0.46	0.19	1.13
Dexa vs Placebo/ga=31 and time=14	31	14	0.46	0.18	1.16
Dexa vs Placebo/ga=31 and time=15	31	15	0.46	0.17	1.21
Dexa vs Placebo/ga=31 and time=16	31	16	0.46	0.16	1.30
Dexa vs Placebo/ga=31 and time=17	31	17	0.46	0.15	1.42
Dexa vs Placebo/ga=31 and time=18	31	18	0.47	0.13	1.61
Dexa vs Placebo/ga=31 and time=19	31	19	0.48	0.12	1.87
Dexa vs Placebo/ga=31 and time=20	31	20	0.49	0.11	2.24
Dexa vs Placebo/ga=31 and time=21	31	21	0.50	0.09	2.78
Dexa vs Placebo/ga=31 and time=22	31	22	0.53	0.08	3.54
Dexa vs Placebo/ga=31 and time=23	31	23	0.55	0.07	4.66
Dexa vs Placebo/ga=31 and time=24	31	24	0.58	0.05	6.32
Dexa vs Placebo/ga=31 and time=25	31	25	0.62	0.04	8.82
Dexa vs Placebo/ga=31 and time=26	31	26	0.66	0.03	12.66
Dexa vs Placebo/ga=31 and time=27	31	27	0.71	0.03	18.69
Dexa vs Placebo/ga=31 and time=28	31	28	0.77	0.02	28.37
Dexa vs Placebo/ga=32 and time=0	32	0	1.08	0.75	1.55
Dexa vs Placebo/ga=32 and time=1	32	1	0.97	0.72	1.32
Dexa vs Placebo/ga=32 and time=2	32	2	0.89	0.65	1.22
Dexa vs Placebo/ga=32 and time=3	32	3	0.81	0.56	1.19
Dexa vs Placebo/ga=32 and time=4	32	4	0.75	0.48	1.19
Dexa vs Placebo/ga=32 and time=5	32	5	0.70	0.41	1.20
Dexa vs Placebo/ga=32 and time=6	32	6	0.65	0.36	1.20
Dexa vs Placebo/ga=32 and time=7	32	7	0.62	0.31	1.21
Dexa vs Placebo/ga=32 and time=8	32	8	0.58	0.28	1.21
Dexa vs Placebo/ga=32 and time=9	32	9	0.56	0.26	1.21
Dexa vs Placebo/ga=32 and time=10	32	10	0.54	0.24	1.21
Dexa vs Placebo/ga=32 and time=11	32	11	0.52	0.22	1.20
Dexa vs Placebo/ga=32 and time=12	32	12	0.51	0.21	1.21
Dexa vs Placebo/ga=32 and time=13	32	13	0.50	0.20	1.23
Dexa vs Placebo/ga=32 and time=14	32	14	0.49	0.19	1.26
Dexa vs Placebo/ga=32 and time=15	32	15	0.49	0.18	1.31
Dexa vs Placebo/ga=32 and time=16	32	16	0.49	0.17	1.40
Dexa vs Placebo/ga=32 and time=17	32	17	0.49	0.16	1.54
Dexa vs Placebo/ga=32 and time=18	32	18	0.50	0.14	1.73
Dexa vs Placebo/ga=32 and time=19	32	19	0.51	0.13	2.01
Dexa vs Placebo/ga=32 and time=20	32	20	0.52	0.11	2.41
Dexa vs Placebo/ga=32 and time=21	32	21	0.54	0.10	2.98
Dexa vs Placebo/ga=32 and time=22	32	22	0.56	0.08	3.79
Dexa vs Placebo/ga=32 and time=23	32	23	0.59	0.07	4.99
Dexa vs Placebo/ga=32 and time=24	32	24	0.62	0.06	6.75
Dexa vs Placebo/ga=32 and time=25	32	25	0.66	0.05	9.42
Dexa vs Placebo/ga=32 and time=26	32	26	0.70	0.04	13.51

Dexa vs Placebo/ga=32 and time=27	32	27	0.76	0.03	19.93
Dexa vs Placebo/ga=32 and time=28	32	28	0.82	0.02	30.24
Dexa vs Placebo/ga=33 and time=0	33	0	1.15	0.75	1.76
Dexa vs Placebo/ga=33 and time=1	33	1	1.04	0.71	1.52
Dexa vs Placebo/ga=33 and time=2	33	2	0.95	0.64	1.40
Dexa vs Placebo/ga=33 and time=3	33	3	0.87	0.56	1.36
Dexa vs Placebo/ga=33 and time=4	33	4	0.80	0.48	1.35
Dexa vs Placebo/ga=33 and time=5	33	5	0.75	0.41	1.34
Dexa vs Placebo/ga=33 and time=6	33	6	0.70	0.36	1.35
Dexa vs Placebo/ga=33 and time=7	33	7	0.66	0.32	1.35
Dexa vs Placebo/ga=33 and time=8	33	8	0.62	0.29	1.34
Dexa vs Placebo/ga=33 and time=9	33	9	0.60	0.27	1.34
Dexa vs Placebo/ga=33 and time=10	33	10	0.57	0.25	1.33
Dexa vs Placebo/ga=33 and time=11	33	11	0.55	0.23	1.33
Dexa vs Placebo/ga=33 and time=12	33	12	0.54	0.22	1.34
Dexa vs Placebo/ga=33 and time=13	33	13	0.53	0.21	1.35
Dexa vs Placebo/ga=33 and time=14	33	14	0.52	0.20	1.38
Dexa vs Placebo/ga=33 and time=15	33	15	0.52	0.19	1.44
Dexa vs Placebo/ga=33 and time=16	33	16	0.52	0.18	1.54
Dexa vs Placebo/ga=33 and time=17	33	17	0.52	0.16	1.68
Dexa vs Placebo/ga=33 and time=18	33	18	0.53	0.15	1.89
Dexa vs Placebo/ga=33 and time=19	33	19	0.54	0.13	2.18
Dexa vs Placebo/ga=33 and time=20	33	20	0.56	0.12	2.61
Dexa vs Placebo/ga=33 and time=21	33	21	0.58	0.10	3.22
Dexa vs Placebo/ga=33 and time=22	33	22	0.60	0.09	4.10
Dexa vs Placebo/ga=33 and time=23	33	23	0.63	0.07	5.37
Dexa vs Placebo/ga=33 and time=24	33	24	0.66	0.06	7.26
Dexa vs Placebo/ga=33 and time=25	33	25	0.70	0.05	10.11
Dexa vs Placebo/ga=33 and time=26	33	26	0.75	0.04	14.49
Dexa vs Placebo/ga=33 and time=27	33	27	0.81	0.03	21.36
Dexa vs Placebo/ga=33 and time=28	33	28	0.88	0.02	32.36
