# Supplementary material for: Effect of dexamethasone on newborn survival at different administration-to-birth intervals: A secondary analysis of the WHO ACTION (Antenatal CorticosTeroids for Improving Outcomes in Preterm Newborn)-I trial
Source: eClinicalMedicine. 2022 Nov 14;53:101744. doi: 10.1016/j.eclinm.2022.101744 (PMC9716334; doi:10.1016/j.eclinm.2022.101744)
Supplement: Caption for Supplementary Material [file mmc3.docx]

**SUPPLEMENTARY APPENDIX**

**Supplementary File S1. Statistical methods for model development**

**S****upplementary File S2. Profilers from multivariate logistic models for newborn outcomes**

- Profiler of P(Neonatal Death).htm
- Profiler of P(Any baby death).htm
- Profiler of P(SRD24h).htm
- Profiler of P(SRDwithin168h).htm

**Supplementary Figure S1. Distribution of administration-to-birth interval by trial arm**

**Chi-square**

**Supplementary Figure S2. Number of births and number of neonatal deaths by week of gestational age at birth**

**Supplementary Figure S3. Relative risks of neonatal mortality in preterm infants exposed to dexamethasone compared to placebo, by administration-to-birth intervals (from 0 through 24 hours), at different gestational ages at first administration**

**Supplementary Figure S4. Relative risks of and stillbirth or neonatal mortality (any baby death) in preterm infants exposed to dexamethasone compared to placebo, by administration-to-birth intervals (from 0 through 28 days), at different gestational ages at first administration**

**Supplementary Figure S5. Relative risks of severe respiratory distress at 24 h in preterm infants exposed to dexamethasone compared to placebo, by administration-to-birth intervals (from 0 through 28 days), at different gestational ages at first administration**

**Supplementary Figure S6. Relative risks of severe respiratory distress at 168 h in preterm infants exposed to dexamethasone compared to placebo, by administration-to-birth intervals (from 0 through 28 days), at different gestational ages at first administration**

**Supplementary Table 1a. Neonatal death (0 to 28 days)**

**Supplementary Table 1b. Neonatal death (0 to 24 hours)**

**Supplementary Table 1c. Any baby death (0 to 28 days)**

**Supplementary Table 1d. Severe respiratory distress at 24 hours (0 to 28 days)**

**Supplementary Table 1e. Severe respiratory distress at 7 days (0 to 28 days)**
