# Supplementary material for: Effect of dexamethasone on newborn survival at different administration-to-birth intervals: A secondary analysis of the WHO ACTION (Antenatal CorticosTeroids for Improving Outcomes in Preterm Newborn)-I trial
Source: eClinicalMedicine. 2022 Nov 14;53:101744. doi: 10.1016/j.eclinm.2022.101744 (PMC9716334; doi:10.1016/j.eclinm.2022.101744)
Supplement: Profiler of P_spl_Neonatal Death_spl_.htm [file mmc5.zip › eclinm_101744_mmc5.htm]

Profiler of P(Neonatal Death)
